# RPGRIP1L is required for stabilizing epidermal keratinocyte adhesion through regulating desmoglein endocytosis

**DOI:** 10.1371/journal.pgen.1007914

**Published:** 2019-01-28

**Authors:** Yeon Ja Choi, Christine Laclef, Ning Yang, Abraham Andreu-Cervera, Joshua Lewis, Xuming Mao, Li Li, Elizabeth R. Snedecor, Ken-Ichi Takemaru, Chuan Qin, Sylvie Schneider-Maunoury, Kenneth R. Shroyer, Yusuf A. Hannun, Peter J. Koch, Richard A. Clark, Aimee S. Payne, Andrew P. Kowalczyk, Jiang Chen

**Affiliations:** 1 Department of Pathology, Stony Brook University, Stony Brook, NY, United States of America; 2 Sorbonne Université, CNRS UMR7622, Inserm U1156, IBPS–Laboratoire de Biologie du Développement, Paris, France; 3 Department of Cell Biology, Emory University School of Medicine, Atlanta, GA, United States of America; 4 Department of Dermatology, University of Pennsylvania, Philadelphia, PA, United States of America; 5 Department of Dermatology, Peking Union Medical College Hospital, Beijing, China; 6 Department of Pharmacology, Stony Brook University, Stony Brook, NY, United States of America; 7 Institute of Laboratory Animal Science, Chinese Academy of Medical Science; and Comparative Medical Center, Peking Union Medical College, Beijing, China; 8 Department of Medicine and Cancer Center, Stony Brook University, Stony Brook, NY, United States of America; 9 Department of Dermatology and Center for Regenerative Medicine, University of Colorado Denver, Aurora, CO, United States of America; 10 Department of Dermatology, Stony Brook University, Stony Brook, NY, United States of America; University of Bradford, UNITED KINGDOM

## Abstract

Cilia-related proteins are believed to be involved in a broad range of cellular processes. Retinitis pigmentosa GTPase regulator interacting protein 1-like (RPGRIP1L) is a ciliary protein required for ciliogenesis in many cell types, including epidermal keratinocytes. Here we report that RPGRIP1L is also involved in the maintenance of desmosomal junctions between keratinocytes. Genetically disrupting the *Rpgrip1l* gene in mice caused intraepidermal blistering, primarily between basal and suprabasal keratinocytes. This blistering phenotype was associated with aberrant expression patterns of desmosomal proteins, impaired desmosome ultrastructure, and compromised cell-cell adhesion *in vivo* and *in vitro*. We found that disrupting the *RPGRIP1L* gene in HaCaT cells, which do not form primary cilia, resulted in mislocalization of desmosomal proteins to the cytoplasm, suggesting a cilia-independent function of RPGRIP1L. Mechanistically, we found that RPGRIP1L regulates the endocytosis of desmogleins such that *RPGRIP1L*-knockdown not only induced spontaneous desmoglein endocytosis, as determined by AK23 labeling and biotinylation assays, but also exacerbated EGTA- or pemphigus vulgaris IgG-induced desmoglein endocytosis. Accordingly, inhibiting endocytosis with dynasore or sucrose rescued these desmosomal phenotypes. Biotinylation assays on cell surface proteins not only reinforced the role of RPGRIP1L in desmoglein endocytosis, but also suggested that RPGRIP1L may be more broadly involved in endocytosis. Thus, data obtained from this study advanced our understanding of the biological functions of RPGRIP1L by identifying its role in the cellular endocytic pathway.

## Author summary

The desmosome is a type of intercellular junction, essential for cells to adhere to one another. Abnormalities in desmosomes can cause disorders in the hair, skin, and heart, some of which are severe or even fatal. Here, we discovered that RPGRIP1L, a protein known to regulate cilia formation and function, is essential for stabilizing desmosomes of skin keratinocytes. Specifically, suppressing the *Rpgrip1l* gene in mice or in keratinocytes disrupted the ultrastructure of desmosomes, and compromised cell-cell adhesion *in vivo* and *in vitro*. We found that knocking down RPGRIP1L in keratinocytes aberrantly accelerated the internalization of cell membrane desmogleins, key desmosomal cadherins. Inhibiting endocytosis effectively rescued these phenotypes. Biotinylation assays confirmed that desmogleins are likely the primary targets of RPGRIP1L. Interestingly, membrane proteins that are not directly associated with the desmosomes were also found to be internalized in *RPGRIP1L*-knockdown cells, raising the possibility that RPGRIP1L might regulate endocytosis more broadly. Findings from this study not only identified RPGRIP1L as a regulator of the desmosomes, but also expanded our understanding of cilia-related proteins in the formation of the desmosomal junctions.

## Introduction

Retinitis pigmentosa GTPase regulator interacting protein 1-like (RPGRIP1L, also known as NPHP8, MKS5, KIAA1005, or Ftm in mouse) is a transition zone protein that has important roles in regulating cilia formation and function [1–5]. Mutations in the *RPGRIP1L* gene cause Joubert syndrome (JBTS) and Meckel syndrome (MKS) [6,7], two severe ciliopathies that are characterized by central nervous system malformation, cystic kidneys, polydactyly, retinal degeneration, and retinal dystrophy [8]. RPGRIP1L participates in the assembly of the ciliary transition zone, autophagy, and activation of the ciliary proteasome [9], whereas mutant RPGRIP1L interferes with ciliary functions, leading to dysplasia of affected organs [6,7,10].

In the skin, *RPGRIP1L* is essential for hair follicle morphogenesis by regulating primary cilia formation and hedgehog signaling [11]. Interestingly, *RPGRIP1L* is also expressed in interfollicular epidermal keratinocytes, many of which are not ciliated [12], suggesting that RPGRIP1L may exert cilia-independent functions in the skin.

Desmosomes are anchoring junctions that are essential for functionalities of tissues that are subjected to constant mechanical stress, such as the skin and the heart. Desmosomal junctions are composed of transmembrane cadherins, desmogleins and desmocollins, and cytoplasmic proteins, including junction plakoglobin (JUP), plakophilins, and desmoplakin (DSP) [13,14]. The adhesion function of desmosomal junctions is dependent on the intercellular anchorage of desmogleins and desmocollins.

The assembly and disassembly of the desmosomes is highly dynamic, and intercalates with cellular events associated with the regulation of the cytoskeleton, intracellular trafficking, ubiquitination, and molecular signaling [13]. Forward and reverse genetic studies continue to uncover new players involved in the formation of the desmosomes, which collectively contribute to the establishment of a comprehensive regulatory network of desmosome assembly and homeostasis.

Mutations in genes encoding desmosomal proteins can cause a range of heritable disorders that affect the skin, hair, and heart, such as monilethrix, woolly hair, palmoplantar keratoderma, and arrhythmogenic right ventricular cardiomyopathy [15–19]. Moreover, disruption of desmosomal junctions by autoantibodies can cause pemphigus, a family of devastating autoimmune disorders characterized by severe intraepithelial blistering in the skin or mucous membranes [20,21]. Loss of desmosomal proteins has, at least in some cases, been linked to cancer development or progression [20,22]. Understanding the cellular and molecular mechanisms underlying the assembly and disassembly of desmosomal junctions is important for the understanding of the pathogenesis of desmosome-related disorders.

In this study, we uncovered a previously unknown function of RPGRIP1L in the formation of the desmosomal junctions. We found that disrupting the *Rpgrip1l* gene in mice or keratinocyte cell lines resulted in desmosomal abnormalities that are associated with aberrant internalization of desmogleins. These findings revealed RPGRIP1L as a regulator of desmosome formation and function, and suggested a broader role of RPGRIP1L in the assembly of cellular organelles, including the ciliary transitional zone and the desmosome.

## Results

### Intraepidermal blistering in *Rpgrip1l*^*–/–*^mice

**Fig 1:**
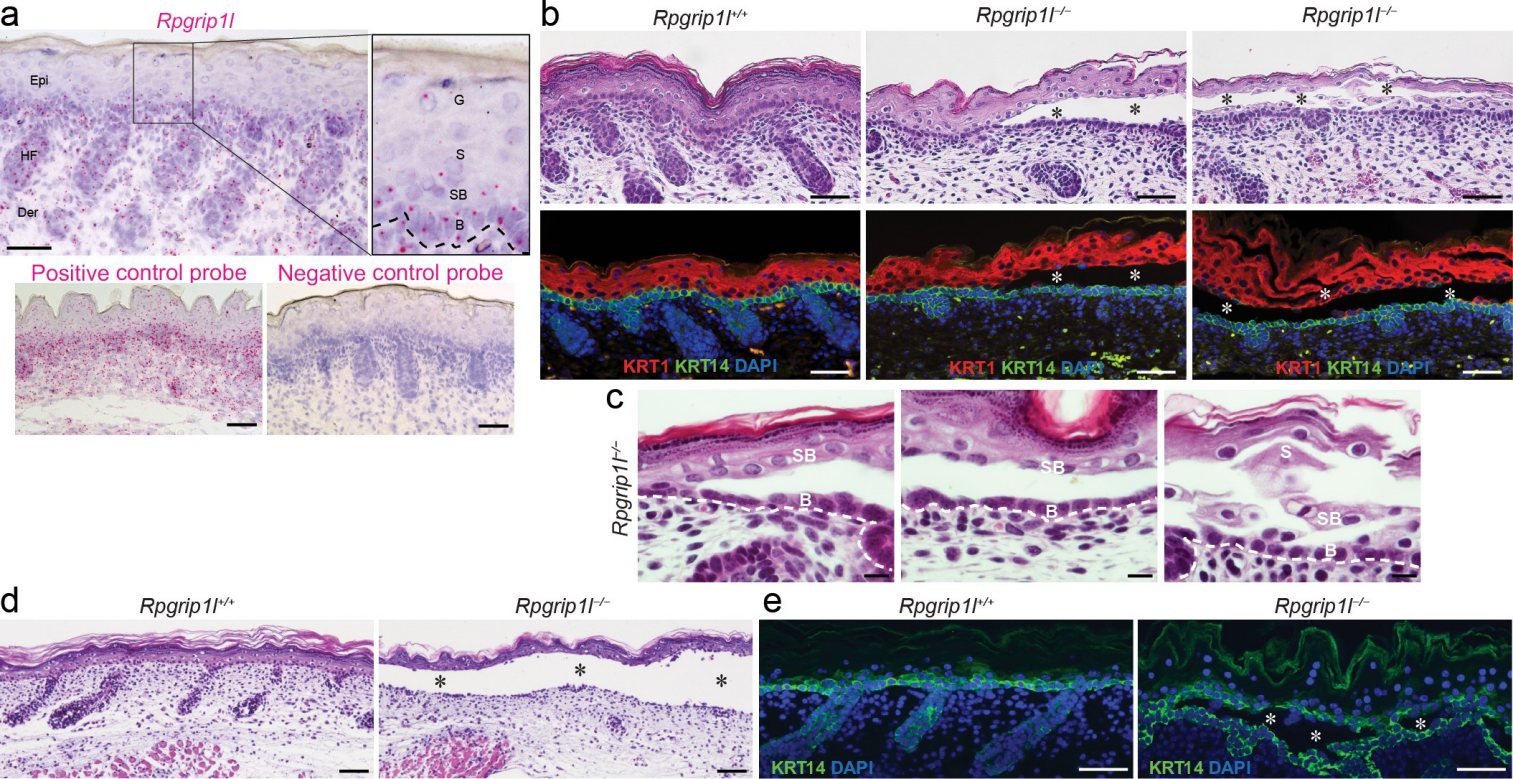
Intraepidermal blistering in *Rpgrip1l*^-/-^ skin. (**a**) *In situ* hybridization of *Rpgrip1l* in the dorsal skin of E18.5 wild-type mouse. *Rpgrip1l* (pink dot) is expressed in the epidermis (Epi), dermis (Der), and hair follicles (HF). Dotted line represents basement membrane. B, basal keratinocyte; SB, suprabasal keratinocyte; S, spinous keratinocyte; G, granular keratinocyte. Positive and negative control probes detect the mouse *POLR2A* or bacterial dapB gene, respectively. (**b**) Hematoxylin and eosin (H&E) staining and immunofluorescence labeling of KRT14 (green) and KRT1 (red) in dorsal skins of E18.5 control (*Rpgrip1*^+/+^) and homozygous mutants (*Rpgrip1l*^-/-^). Nuclei were labeled with DAPI (blue). Asterisks indicate intraepidermal blisters. (**c**) High power H&E images to demonstrate details of the blistering region. B, basal keratinocyte; SB, suprabasal keratinocyte; S, spinous keratinocyte. (**d** and **e**) H&E staining (d) and immunofluorescence labeling of KRT14 (green, e) of organotypic skin explants from E18.5 control (*Rpgrip1l*^+/+^, n = 8) and homozygous (*Rpgrip1l*^–/–^, n = 7) embryos at day 2. Nuclei were labeled with DAPI (blue). Asterisks indicate intraepidermal blisters. Scale bar, 25 μm in (a), 100 μm in (b), 10 μm in (c), 50 μm in (d, e).

*Rpgrip1l* is ubiquitously expressed in the skin, including the epidermis, dermis, and hair follicles [11]. In mouse epidermis, the *Rpgrip1l* transcript, as determined by *in situ* hybridization, is consistently expressed in basal epidermal keratinocytes and, to a lesser extent, in spinous and granular cells (Fig 1A). The RPGRIP1L protein is enriched between the basal body (marked by gamma-tubulin, γ-Tub) and ciliary axoneme (marked by acetylated α-tubulin, α-Tub) of ciliated basal keratinocytes (S1A Fig), or enriched at the centrioles of unciliated keratinocytes (S1E Fig), but below detection in *Rpgrip1l* knockout (*Rpgrip1l*^*–/–*^) epidermis (S1I Fig). Since differentiated epidermal keratinocytes are rarely ciliated [12,23,24], the widespread expression pattern of *Rpgrip1l* in the epidermis raised the possibility that *Rpgrip1l* performs functions beyond regulating ciliogenesis and ciliary functions.

Indeed, skins of 50% of E18.5 *Rpgrip1l*^*–/–*^embryos (n = 16) exhibited focal intraepidermal blistering, predominantly between basal and suprabasal keratinocytes, marked by KRT14 and KRT1, respectively (Fig 1B, middle panels). In severe cases, cell-cell detachments could also be observed in the spinous and granular layers (Fig 1B, right panels). To further explore this relatively sporadic blistering phenotype, which was not sufficiently characterized in a previous study [11], and to circumvent perinatal lethality associated with severe developmental abnormalities in *Rpgrip1l*^*–/–*^mutants, including exencephaly and ventricular septal defects [2,25], we cultured skins isolated from E18.5 embryos. Organ-cultured *Rpgrip1l*^*–/–*^skins exhibited widespread blistering between the basal and suprabasal layers (Fig 1D and 1E), suggesting that blistering is progressive as the skin becomes mature. Histologically, this intraepidermal blistering phenotype was not associated with discernable cytolysis of keratinocytes, detachment of the basement membrane (Fig 1C), or apoptosis (S2 Fig). Thus, these findings suggest that the blistering phenotype observed in *Rpgrip1l*^*–/–*^skin may be associated with abnormalities in keratinocyte adhesion.

### RPGRIP1L is likely associated with desmosomal adhesion in the skin

**Fig 2:**
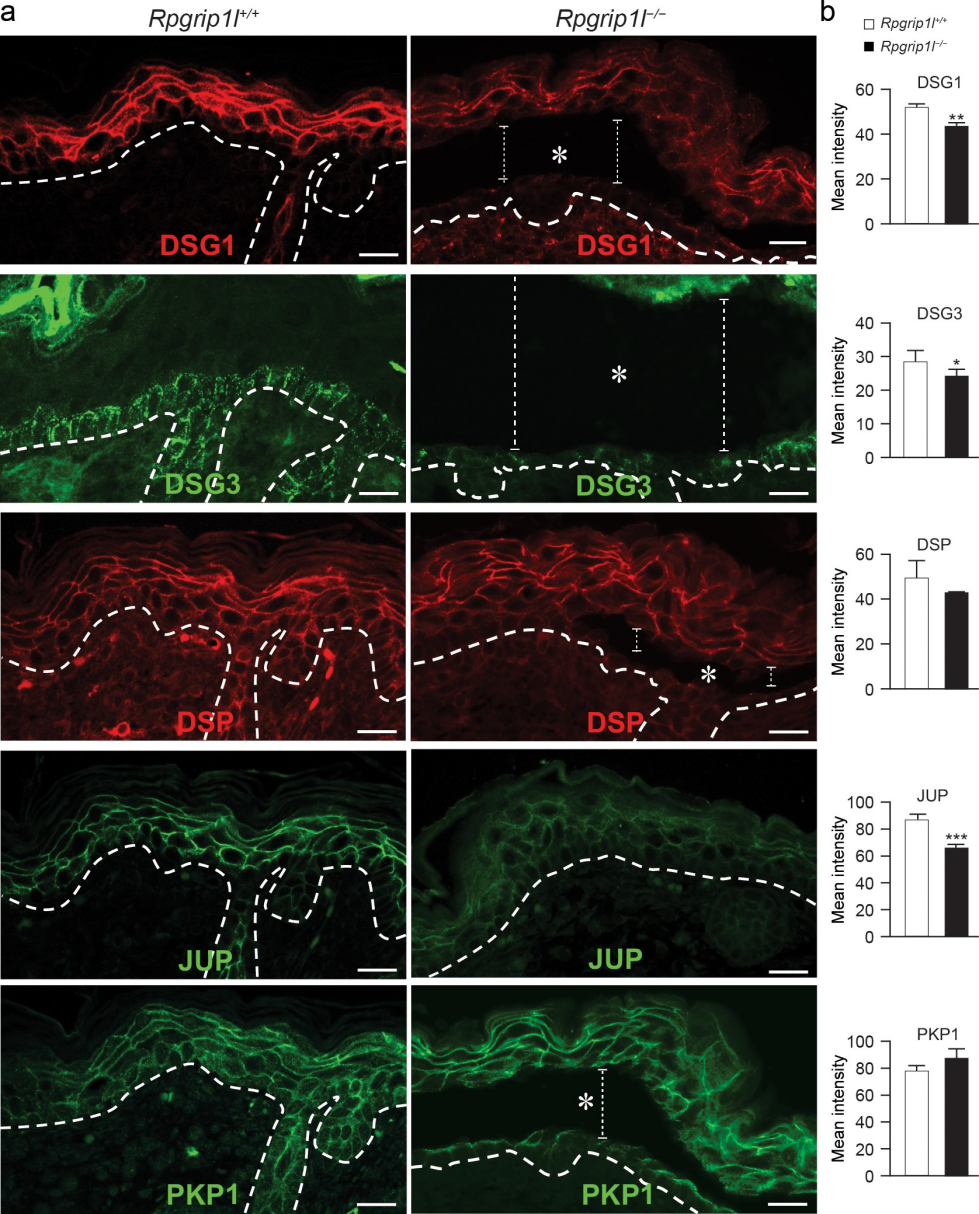
Expression patterns of desmosomal proteins in *Rpgrip1l*^-/-^ skin. (**a**) Immunofluorescence labeling of desmosomal proteins in dorsal skins of E18.5 control (*Rpgrip1l*^+/+^) and homozygous (*Rpgrip1l*^-/-^) mutants. Dotted lines illustrate epidermal-dermal junction. Asterisks and vertical lines indicate blisters between basal and suprabasal cells. (**b**) Quantification of the mean fluorescence intensity of corresponding panels in (a) (2 regions per specimen, n ≥ 3 mice per group;* *P* < 0.05, ** *P* < 0.01 *** *P* < 0.001; Student’s *t*-test). Scale bars, 20 μm.

The desmosomal junctions play essential roles in epidermal adhesion and were, therefore, examined in *Rpgrip1l*^*–/–*^mutants. Immunofluorescence labeling revealed reduced expression of desmoglein 1 (DSG1), desmoglein 3 (DSG3), JUP, and desmocollin 1 (DSC1) in *Rpgrip1l*^*–/–*^skin (Fig 2 and S3 Fig). Specifically, these proteins were diffusely localized to the cytoplasm of keratinocytes of *Rpgrip1l*^*–/–*^skin, in contrast to the predominant plasma membrane localization in control skins (Fig 2 and S3 Fig). The expression patterns of DSP and desmocollin 2 and 3 (DSC2/3) appeared slightly perturbed in *Rpgrip1l*^*–/–*^skin, whereas the expression of plakophilin 1 (PKP1) did not seem to change, as judged by immunofluorescence microscopy (Fig 2 and S3 Fig).

**Fig 3:**
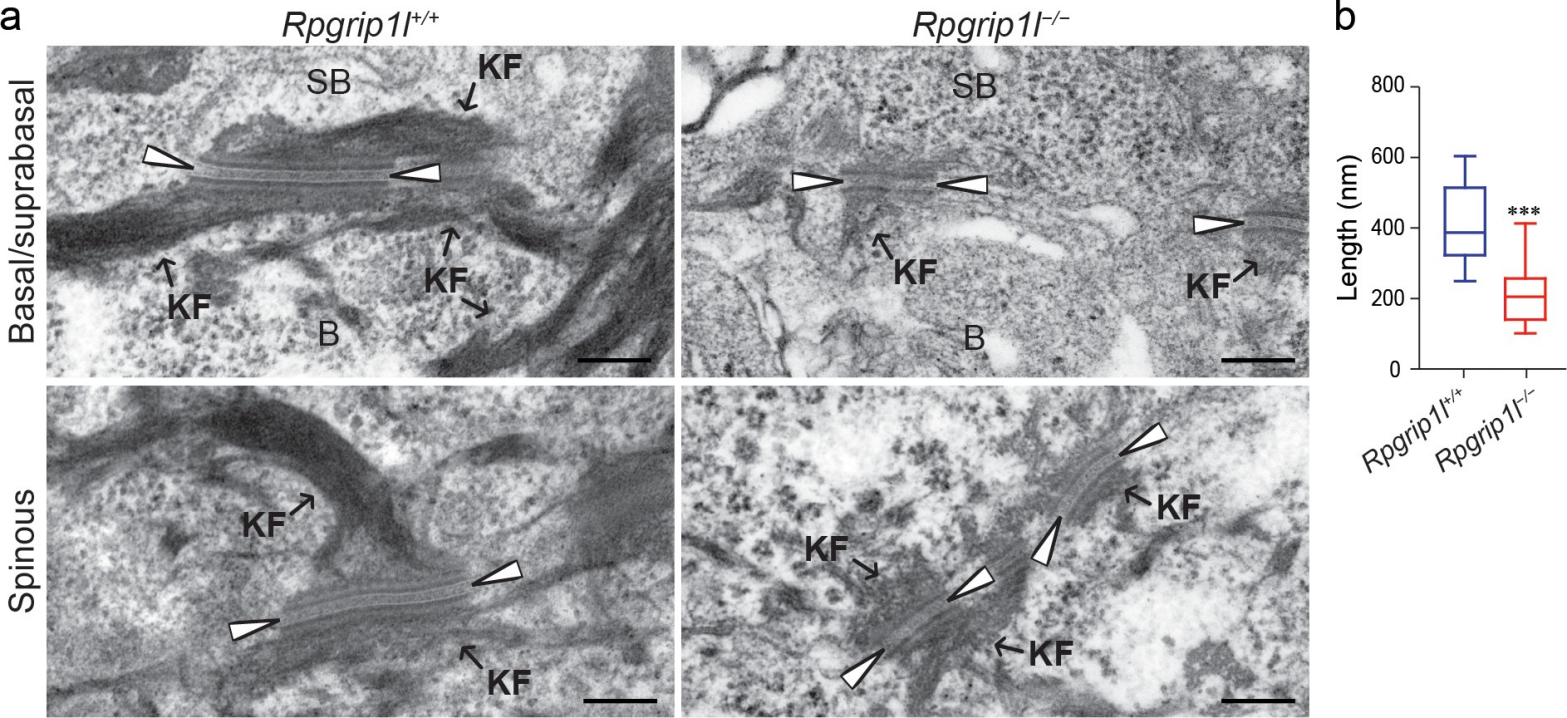
Ultrastructure of desmosomes in *Rpgrip1l*^-/-^ mice. (**a**) Transmission electron microscopy micrographs of desmosomes between basal and suprabasal keratinocytes (Basal/suprabasal), and between spinous keratinocytes (Spinous) in E18.5 control (*Rpgrip1l*^+/+^) and homozygous (*Rpgrip1l*^-/-^) mutant (*n* = 7 mice). Arrowheads point to the electron dense midline. B, basal keratinocytes; SB, suprabasal keratinocytes; KF, keratin filaments. (**b**) Quantification of the length of desmosomes between basal and suprabasal keratinocytes of control and *Rpgrip1l*^-/-^ mutants (*n* ≥ 15 desmosomes; 3 mice per group; *** *P* < 0.001; Student’s *t*-test). Scale bars, 200 nm.

Transmission electron microscopy (TEM) revealed that desmosomes between basal and suprabasal keratinocytes were significantly shorter in *Rpgrip1l*^*–/–*^mutants (Fig 3A and 3B). Moreover, in *Rpgrip1l*^*–/–*^skin, the electron dense midline of desmosomes was less prominent or invisible, the keratin filament attachment was reduced, and the outer electron dense plaque appeared less dense or disorganized (Fig 3A). Similar defects were occasionally observed between spinous keratinocytes (Fig 3A). These findings demonstrated that the blistering phenotype in *Rpgrip1l*^*–/–*^skin is correlated with abnormalities in the desmosomal junctions. Interestingly, adherens junctions, as assessed by immunofluorescence labeling of E-cadherin (CDH1), α-catenin (CTNNA1), and β-catenin (CTNNB1), exhibited only subtle perturbations in *Rpgrip1l*^*–/–*^skin (S3 and S4 Figs). Taken together, these data suggest that RPGRIP1L may be required for keratinocyte adhesion primarily through regulating desmosomal junction formation *in vivo*.

### RPGRIP1L is essential for keratinocyte adhesion *in vitro*

**Fig 4:**
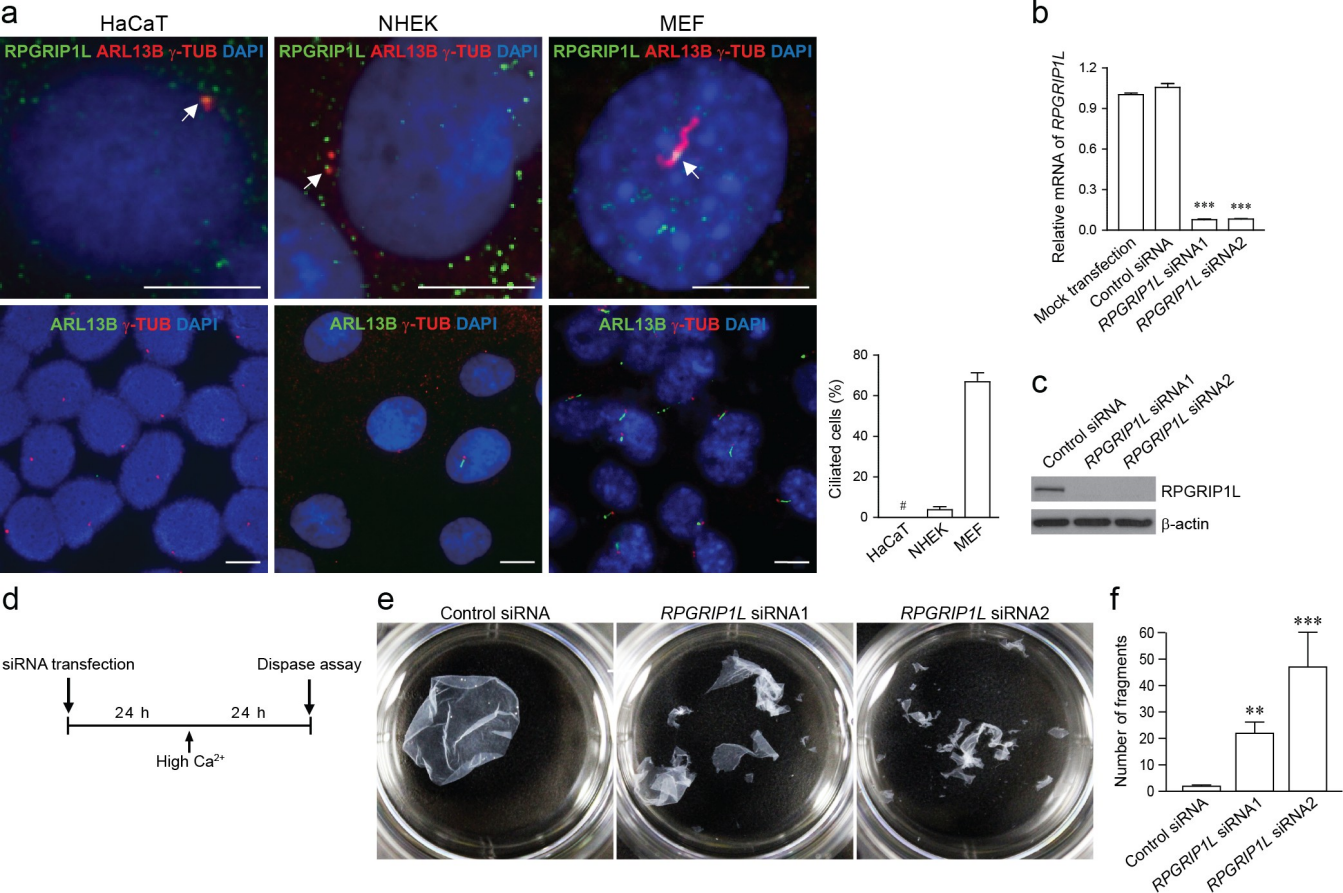
*RPGRIP1L*-knockdown in keratinocytes compromises cell-cell adhesion. (**a**) Immunofluorescence labeling of RPGRIP1L, primary cilia (ARL13B), and basal body/centriole (γ-TUB) in HaCaT cells, normal human epidermal keratinocytes (NHEK), and mouse embryonic fibroblasts (MEFs) at 48 hours after serum-starvation. Nuclei were labeled with DAPI (blue). Arrows point to centrioles or a basal body where RPGRIP1L is enriched. Bar graph represents percentage of ciliated cells (# indicates that cilium was undetectable in HaCaT cells). Scale bar, 10 μm. (**b** and **c**) Verification of *RPGRIP1L*-knockdown by qRT-PCR (b) and western blotting (c) in HaCaT cells. Values were normalized to mock transfection in (b). (**d**) Timeline of the dispase dissociation assay. (**e**) Dispase dissociation assay in control (Control siRNA) and *RPGRIP1L*-knockdown (*RPGRIP1L* siRNA) HaCaT cells. (**f**) Quantification of cell fragments (mean ± SEM, *n* = 3 independent experiments; ** *P* < 0.01; *** *P* < 0.001; Student’s *t*-test).

RPGRIP1L is expressed in many cell types, and is enriched at the base of cilia for its cilia-related functions [1,2,6,7,10,26]. To evaluate the roles of RPGRIP1L in desmosome formation, we first examined the expression pattern of RPGRIP1L in HaCaT cells and normal human epidermal keratinocytes (NHEKs). HaCaT cells do not form primary cilia, and NHEKs rarely form primary cilia (3.4 ± 2.6%) after serum starvation, in comparison to mouse embryonic fibroblasts (MEFs) which do (66.7 ± 12.5%) (Fig 4A). In HaCaT cells and NHEKs, RPGRIP1L is enriched at the centrosomes (marked by γ-TUB) and diffusely distributed in the cytoplasm (Fig 4A). These findings suggest that the potential role of RPGRIP1L in desmosome formation is independent of its role in ciliogenesis in keratinocytes.

We subsequently knocked down the endogenous *RPGRIP1L* gene in HaCaT cells by siRNAs (Fig 4B and 4C). Knockdown cells were then treated with high calcium to allow desmosomal junctions to form, then subjected to dispase dissociation assay as illustrated in Fig 4D. *RPGRIP1L*-knockdown markedly compromised the integrity of the epidermal sheet, resulting in significantly increased fragmentation (Fig 4E and 4F). This experiment indicated that RPGRIP1L is functionally required for cell-cell adhesion of keratinocytes *in vitro*.

### Desmosomal junction is disrupted in *RPGRIP1L*-knockdown keratinocytes

**Fig 5:**
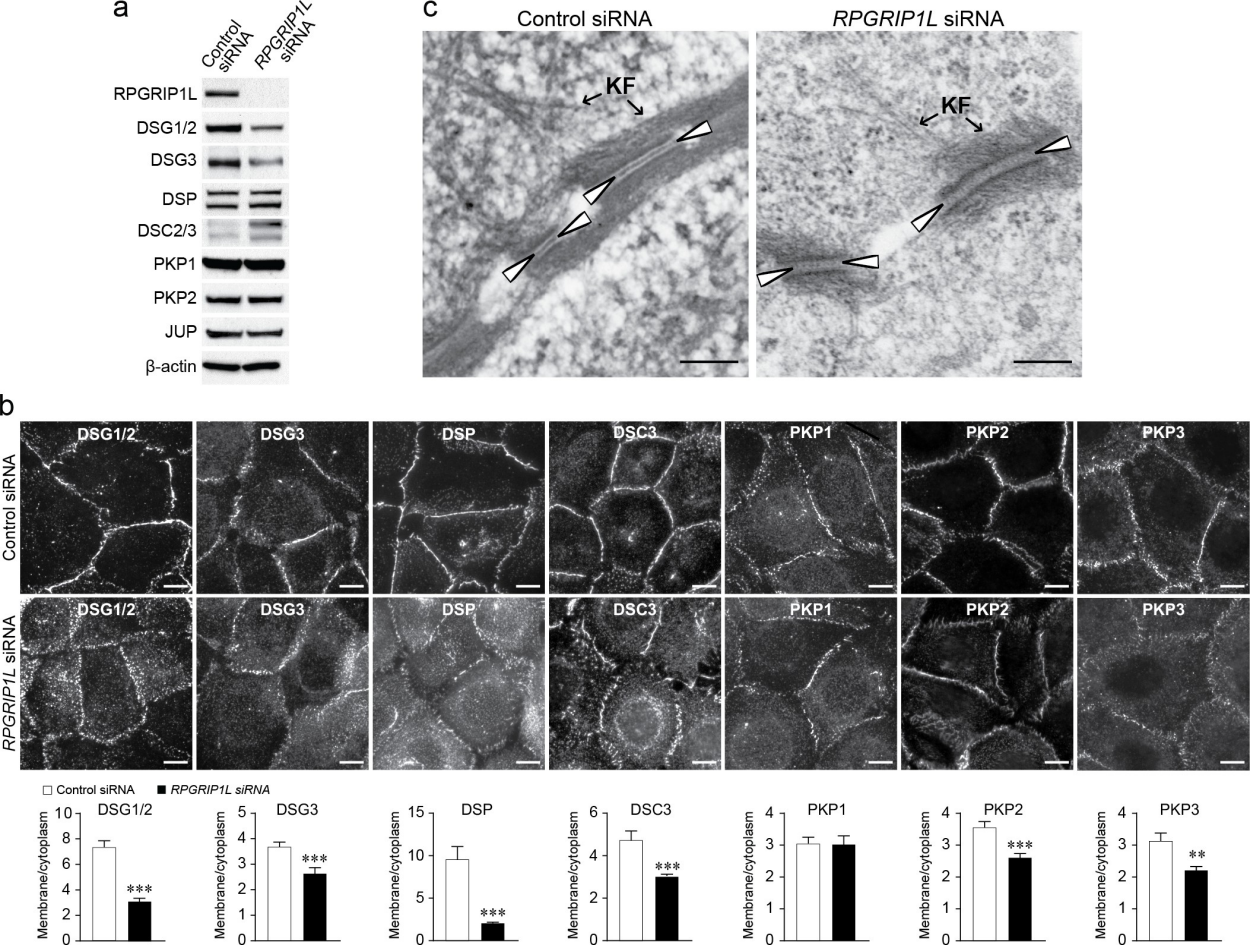
*RPGRIP1L*-knockdown disrupts desmosomes in HaCaT cells. (**a**) Expression of desmosomal proteins in control (Control siRNA) and *RPGRIP1L*-knockdown (*RPGRIP1L* siRNA) HaCaT cells by western blotting. Note that both DSG1 and DSG2 are expressed in HaCaT cells, and detectable by the DSG1/2 antibody. (**b**) Immunofluorescence labeling of desmosomal proteins in control and *RPGRIP1L*-knockdown HaCaT cells. Bar graphs represent plasma membrane/cytoplasm ratios of average pixel intensities of desmosomal proteins (n = 50 cells, ** *P* < 0.01, *** *P* < 0.001; Student’s *t*-test). (**c**) Transmission electron microscopy micrographs of desmosomes in control and *RPGRIP1L*-knockdown HaCaT cells. Arrowheads point to the electron dense midline. KF, keratin filaments. Scale bar, 10 μm in (b), 200 nm in (c).

To further confirm *in vivo* findings, desmosomal junctions were evaluated in *RPGRIP1L*-knockdown HaCaT cells and NHEKs. *RPGRIP1L*-knockdown did not significantly affect cell viability (S5 Fig), but resulted in marked reduction of desmoglein 1 and 2 (DSG1/2) and desmoglein 3 (DSG3) proteins as determined by western blotting (Fig 5A and S6 Fig), but not mRNA (S7 Fig). The protein levels of DSP, PKP1, plakophilin 2 (PKP2), and JUP were unaffected, whereas those of DSC2/3 increased in *RPGRIP1L*-knockdown cells (Fig 5A and S6 Fig). Immunofluorescence labeling demonstrated that the membrane localization of many desmosomal proteins, including DSG1/2 and DSG3, were significantly reduced in *RPGRIP1L*-knockdown cells (Fig 5B). These findings suggest that disrupting *RPGRIP1L* expression in keratinocytes impairs the stability and membrane localization of desmosomal proteins.

At the ultrastructural level, *RPGRIP1L*-knockdown HaCaT cells exhibited desmosomal abnormalities that are similar to those observed *in vivo*, including disrupted midline and reduced keratin attachment (Fig 5C). Taken together, these *in vitro* results further substantiated the role of RPGRIP1L in maintaining structural integrity of the desmosomes.

In contrast, *RPGRIP1L*-knockdown did not result in discernable changes in the expression pattern of intermediate filaments in HaCaT cells as demonstrated by KRT14 immunostaining (S8 Fig).

### Loss of RPGRIP1L induces internalization of the desmogleins

Because disrupting *Rpgrip1l* resulted in consistent changes in the desmogleins under both *in vivo* and *in vitro* conditions, and the blistering phenotype observed in *Rpgrip1l*^*–/–*^skins is similar to what is seen in pemphigus, a severe blistering disorder caused primarily by the disruption of the desmogleins, we focused our investigation on the desmogleins.

**Fig 6:**
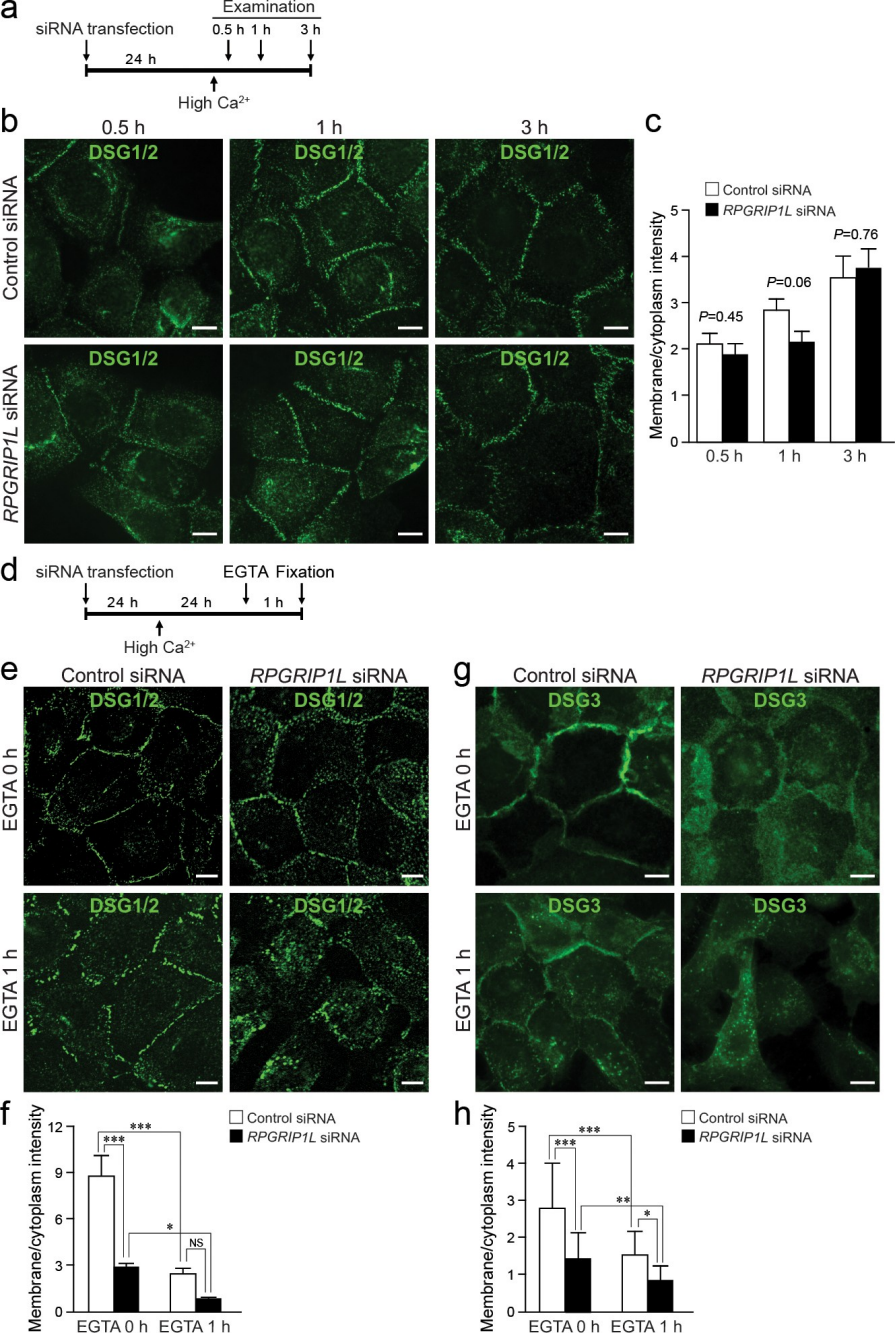
Disrupting *RPGRIP1L* promotes the disassembly of desmosomes. (**a**) Timeline of knockdown and desmosome assembly assay. (**b** and **c**) Immunofluorescence labeling (b) and quantification (c) of DSG1/2 at 0.5, 1, and 3 hours after switching to high calcium media in control (Control siRNA) and *RPGRIP1L*-knockdown (*RPGRIP1L* siRNA) HaCaT cells. (**d**) Timeline of knockdown and desmosome disassembly assay. (**e** and **f**) Immunofluorescence labeling (e) and quantification (f) of DSG1/2 in control and *RPGRIP1L*-knockdown HaCaT cells treated with EGTA. (**g** and **h**) Immunofluorescence labeling (g) and quantification (h) of DSG3 as described for DSG1/2. Quantifications (c, f, and h) represent plasma membrane/cytoplasm ratios of the pixel intensities of DSG1/2 or DSG3 (n = 25 cells, * *P* < 0.05, ** *P* < 0.01, *** *P* < 0.001, NS, not statistically significant, Bonferroni’s *post hoc* tests. Note, when EGTA-treated cells were compared directly, *RPGRIP1L*-knockdown elicited a significant reduction of membrane DSG1/2 in f, *P* = 0.049). Scale bar, 10 μm.

The formation of desmosomes is highly dynamic and can be arbitrarily divided into the assembly and disassembly phases. In HaCaT cells, desmosomes start to assemble when the cells are exposed to high calcium. We found that knocking down *RPGRIP1L* during desmosome assembly (0.5, 1, and 3 hours after shifting to high calcium, as illustrated in Fig 6A) did not impair the accumulation of DSG1/2 to the plasma membrane, as determined by immunofluorescence labeling (Fig 6B and quantification in 6C), suggesting that RPGRIP1L might be dispensable for desmosome assembly. In contrast, in the disassembly assay (as illustrated in Fig 6D), where DSG1/2 and DSG3 were examined 24 hours after calcium switch, in conjunction with 1-hour EGTA treatment to further induce desmosome disassembly, the plasma membrane localization of DSG1/2 or DSG3 was significantly decreased in *RPGRIP1L*-knockdown cells such that DSG1/2 or DSG3 appeared discontinuous along, or in some cases absent from the plasma membrane (Fig 6E and 6G, respectively). Quantifications of membrane and cytoplasmic signal intensity showed that the membrane/cytoplasmic ratio of DSG1/2 or DSG3 was significantly reduced in knockdown cells, a phenotype that was further exacerbated upon EGTA treatment (Fig 6F and 6H, respectively). These findings suggest that loss of RPGRIP1L may cause increased internalization of cell surface desmogleins. This result is consistent with a well-established model in which increased desmoglein endocytosis leads to a decrease in both cell surface and steady-state levels of desmogleins, as seen in Figs 2 and 5 and S6B Fig [27,28].

### Blocking endocytosis is sufficient to rescue aberrant internalization of desmogleins in *RPGRIP1L*-knockdown cells

**Fig 7:**
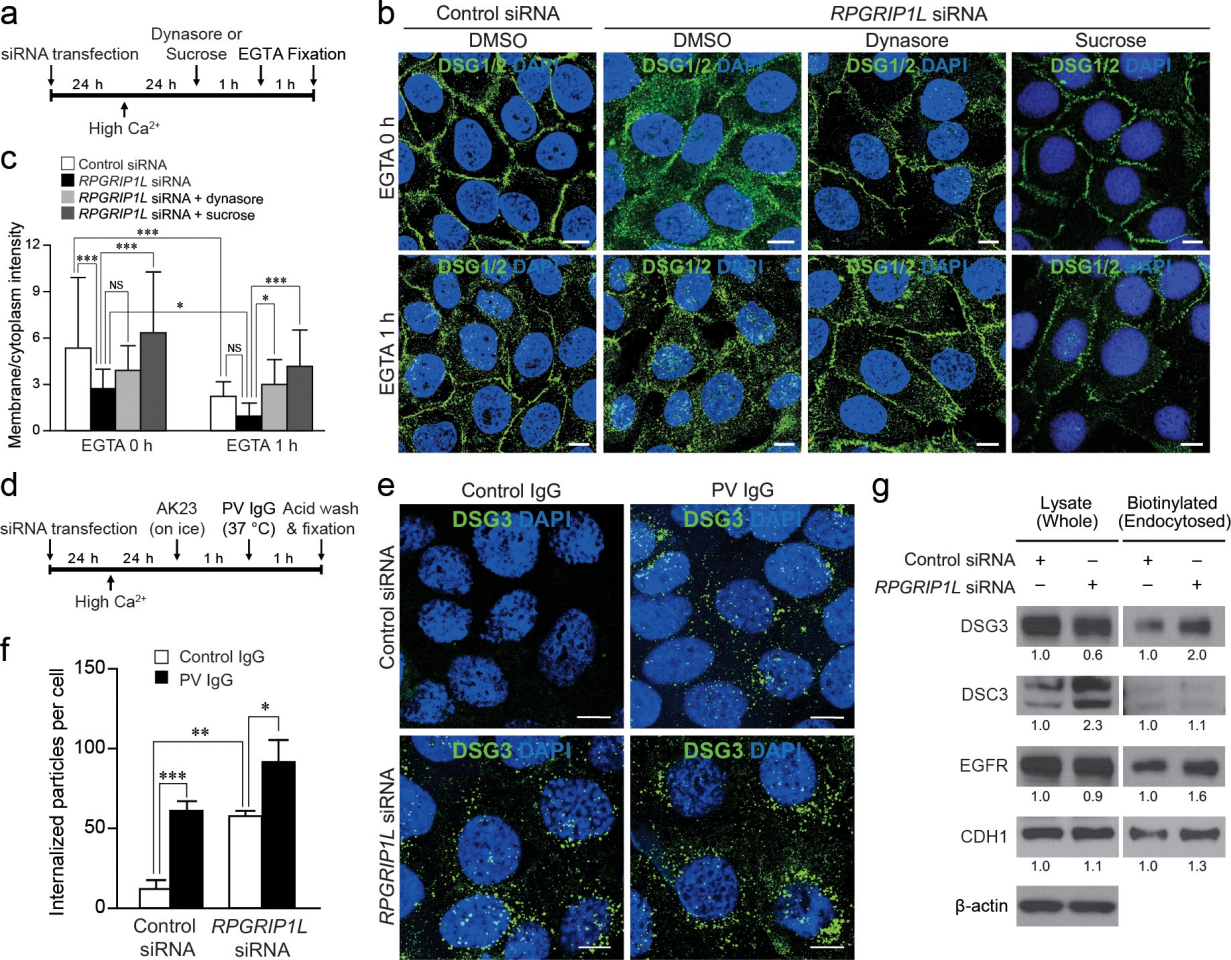
Disrupting *RPGRIP1L* promotes endocytosis of desmogleins in HaCaT cells. (**a**) Timeline of *RPGRIP1L*-knockdown and treatment with dynasore or sucrose, and EGTA. (**b**) Immunofluorescence labeling of DSG1/2 in control (Control siRNA) and *RPGRIP1L*-knockdown (*RPGRIP1L* siRNA) cells treated with dynasore or sucrose, and EGTA. Nuclei were labeled with DAPI (blue). (**c**) Quantification of the ratios of plasma membrane/cytoplasm pixel intensities of DSG1/2 in (b) (n = 25). (**d**) Timeline of the cell surface DSG3 internalization assay carried out with the AK23 antibody. (**e**) Immunofluorescence of internalized DSG3 (labeled by the AK23 antibody) in control and *RPGRIP1L*-knockdown cells treated with PV IgG. Nuclei were labeled with DAPI (blue). (**f**) Quantification of the number of particles (DSG3) per cell (n ≥ 160 cell per group, three independent experiments). (**g**) Detection of cell-surface proteins (DSG3, DSC3, EGFR and CDH1) by western blotting in whole cell lysate and biotinylated (endocytosed) fraction in a biotinylation assay on control and *RPGRIP1L*-knockdown cells. The numbers indicate fold changes relative to control siRNA knockdowns as determined by densitometry. * *P* < 0.05, ** *P* < 0.01, *** *P* < 0.001 (Bonferroni’s *post hoc* tests). Note, when EGTA-treated cells were compared directly, *RPGRIP1L*-knockdown elicited a significant reduction of membrane DSG1/2 in c, P = 0.027. Scale bar, 10 μm.

Desmoglein internalization is mediated by multiple endocytic mechanisms and remains a subject of further investigation [29–37]. Nevertheless, blocking endocytosis could prevent the internalization of desmogleins that are present on the cell surface. Here, we utilize two well-established approaches to blocking endocytosis to determine whether aberrantly accelerated internalization of desmogleins in *RPGRIP1L*-difficient cells is functionally responsible for the loss of membrane desmogleins. One approach was to use dynasore, a specific inhibitor of dynamin GTPase activity [38,39], to suppress endocytosis. Dynasore had been shown capable of stabilizing desmosomal junctions through blocking endocytosis [38]. The other approach was to use hyperosmotic sucrose to suppress endocytosis [40]. Dynasore or sucrose was added 24 hours after shifting to high calcium and 2 hour prior to fixation, as illustrated in Fig 7A.

Pretreating *RPGRIP1L*-knockdown cells with 50 μM dynasore for two hours was sufficient to rescue the increased internalization of DSG1/2, as determined by immunofluorescence labeling (Fig 7B). Specifically, the membrane localization of DSG1/2 in *RPGRIP1L*-knockdown cells was restored to a level comparable to that of control knockdown (Fig 7B, upper panels, and quantifications in c). Furthermore, dynasore treatment also overcame the additive effects of both knockdown- and EGTA-induced DSG1/2 internalization (Fig 7B, lower panels, and quantifications in 7C). Similarly, hyperosmotic sucrose effectively rescued *RPGRIP1L* knockdown-induced DSG1/2 internalization in HaCaT cells, even in the presence of EGTA, as demonstrated by immunofluorescence labeling and quantification (Fig 7B, right panels, and 7C). Collectively, these rescue experiments suggest that elevated endocytosis is functionally responsible for *RPGRIP1L* knockdown-induced internalization of the desmogleins.

### *RPGRIP1L*-knockdown induces internalization of surface DSG3 in a manner similar to pemphigus vulgaris patient-derived (PV) IgG

To directly monitor the endocytosis of desmogleins, we performed an internalization assay, in which the internalized DSG3 can be quantified by labeling DSG3 with AK23, a monoclonal antibody against the extracellular domain of DSG3 [41], in live cells as previously demonstrated [29]. Specifically, as outlined in Fig 7D, AK23 was used to label cell surface DSG3 on ice, one hour prior to shifting to 37°C to trigger internalization (in the presence of control or PV IgG). One hour later, AK23-labeled but not internalized DSG3 was stripped off cell surface through acid wash. Cells were then fixed, and the internalized/AK23-labled DSG3 was detected by immunofluorescence.

In control siRNA-treated cells, control IgG treatment resulted in low levels of internalization of DSG3 (Fig 7E, upper left panel). As expected, PV IgG induced marked internalization of DSG3 (Fig 7E, upper right panel, and quantifications in 7F). Remarkably, *RPGRIP1L*-knockdown also resulted in marked internalization of DSG3 (Fig 7E, lower left panel), an effect as robust as PV IgG, as quantified in Fig 7F, reinforcing prior findings that the loss of RPGRIP1L can be pathogenic. More interestingly, DSG3 internalization was further increased by dual PV IgG and *RPGRIP1L* siRNA treatments (Fig 7E, lower right panel, and quantifications in 7F). This additive effect suggested that loss-of-RPGRIP1L and PV IgG may promote DSG3 endocytosis through distinct mechanisms.

### A potential role of RPGRIP1L in regulating global endocytosis

The above findings established a role of RPGRIP1L in regulating the endocytosis of cell surface desmogleins in epidermal keratinocytes. To determine whether RPGRIP1L might be more broadly involved in endocytosis, we evaluated the steady state-rate of endocytosis of cell-surface proteins by biotinylation assays [42]. First, the level of DSG3 decreased in whole cell lysate of *RPGRIP1L*-knockdown cells (Fig 7G), whereas biotinylated (endocytosed) DSG3 markedly increased in *RPGRIP1L*-knockdown cells (Fig 7G), a finding that is consistent with the above data (Figs 5B, 6G and 7E). The total protein level of DSC3 increased, whereas biotinylated (endocytosed) DSC3 increased marginally in this biotinylation assay (Fig 7G), also consistent with previously obtained data (Fig 5A and 5B).

Next, we examined cell membrane-associated proteins other than desmogleins, specifically epidermal growth factor receptor (EGFR) and CDH1. Despite the comparable total levels of EGFR and CDH1 in control and *RPGRIP1L*-knockdown cells (Fig 7G, left panels), *RPGRIP1L*-knockdown correlated with increased levels of biotinylated (endocytosed) EGFR and CDH1 (Fig 7G, right panels). Although the desmogleins cross-regulate with many cell surface proteins, including EGFR [43,44] and CDH1 [45–47], increased endocytosis of EGFR and CDH1 in association with *RPGRIP1L*-knockdown nevertheless suggests that RPGRIP1L may regulate endocytosis more broadly, which is worthy of future investigation.

Collectively, data obtained from this study suggest that increased endocytosis of desmogleins is primarily responsible for the keratinocyte adhesion defects associated with RPGRIP1L deficiency, thereby establishing a role of RPGRIP1L in stabilizing the desmosomes in skin.

## Discussion

Increasing evidence suggests that cilia-related proteins perform important cellular functions beyond regulating cilia formation or function [48]. In this study, we demonstrated that a ciliary protein, RPGRIP1L, is required for the maintenance of desmosomal junctions through regulating endocytosis of desmogleins in epidermal keratinocytes, thereby extending the functions of cilia-related proteins to cell-cell adhesion. Intriguingly, JBTS and MKS patients, who harbor loss-of-function mutations in the *RPGRIP1L* gene, do not exhibit blistering phenotypes. It is possible that blistering in these patients is underdiagnosed, or that abnormalities in desmosomal junctions exist but are subclinical. It is also plausible that the mutant *RPGRIP1L* gene products in patients retain a certain level of functionality, whereas genetically disrupting the *Rpgrip1l* locus leads to more catastrophic phenotypes in mice, by which blistering was observed. Further understanding the molecular mechanisms through which RPGRIP1L participates in desmosomal junction formation will shed light on how RPGRIP1L performs diverse functions in ciliogenesis and desmosome formation.

RPGRIP1L is highly enriched at the transition zone of cilia, but is also distributed in the cytoplasm and at the plasma membrane [6,49]. Thus, our finding that RPGRIP1L performs functions beyond the cilia is not entirely surprising. Data obtained from this study support a role of RPGRIP1L in stabilizing desmogleins at the plasma membrane, thus qualifying RPGRIP1L as a regulator of desmoglein internalization.

The precise molecular mechanism through which RPGRIP1L regulates desmoglein internalization remains to be uncovered. Without a strong presence at the plasma membrane, it is unlikely that RPGRIP1L interacts with desmosomal proteins at the cell membrane as previously described for Lis1, adducin, and flotillins [50–52]. Rather, in keratinocytes, RPGRIP1L is enriched at the base of the cilium as well as the centrosomes, both hubs for intracellular trafficking. RPGRIP1L may modulate desmoglein internalization by interacting with cytoplasmic regulators of the desmosomes, or through signaling, such as PKCα [37,38,53–56] or p38/MAPK [30,57–59]. Because keratinocyte proliferation, differentiation, and apoptosis are not markedly impaired in RPGRIP1L-deficient cells, the mechanism through which RPGRIP1L regulates desmoglein internalization is likely to be specific to the desmosomal regulatory network.

It is well established that RPGRIP1L physically interacts with NPHP4 during cilia formation or function [7,49,60]. NPHP4 is not only required for proper cilia formation, but also implicated in the formation of tight junctions [61]. In the current study, we provided evidence that RPGRIP1L is required for the proper formation and function of the desmosomal junctions, primarily through regulating endocytosis of desmogleins. Interestingly, we also observed, albeit inconsistently, changes in components of adherens junctions, including CDH1, CTNNA1, and CTNNB1. Considering the well-documented cross-talk between these intercellular anchoring junctions [62,63], we postulate that the desmosomes are the primary targets of RPGRIP1L in keratinocytes. This evidence nevertheless raised the possibility that RPGRIP1L may be more broadly involved in cell-cell junctions through the RPGRIP1L-NPHP4 protein complex [7,49]. The functional requirement of NPHP4 or the RPGRIP1L-NPHP4 protein complex in desmosome formation remains to be determined.

The desmogleins were consistently the most markedly endocytosed proteins. In unbiased biotinylation assays, elevated internalization of DSG3 was also correlated with increased endocytosis of other cell surface proteins in *RPGRIP1L*-knockdown cells. It remains to be determined whether this is a mere correlation, or whether RPGRIP1L is functionally associated with endocytosis of other cell surface molecules. Generalization of the potential role of RPGRIP1L in internalization of membrane molecules may further our understanding of endocytosis and recycling in both ciliated and unciliated cells. Given that the current knowledge of the molecular functions of RPGRIP1L is limited to its role in proteasome activity and autophagy at the base of the cilium [26,64], it is plausible that RPGRIP1L also participates in endocytosis through regulating protein degradation, a potential mechanism that needs to be further dissected.

The current study focused on the desmogleins, whose expression levels and membrane localizations were found to be consistently compromised *in vivo* and *in vitro*, mimicking key pathological features observed in pemphigus. We found that components of the desmosome were not equally affected in *Rpgrip1l*^*–/–*^skin and *RPGRIP1L*-knockdown cells. The levels of most other desmosomal proteins remained unchanged with the exception of DSC2/3 increasing, whereas PKP1 did not exhibit increased internalization in *RPGRIP1L*-knockdown cells. It is plausible that the increased DSC2/3 might have helped PKP1 to associate with the cell membrane in *RPGRIP1L*-deficient cells. In light of these findings, we postulate that desmogleins may be the primary targets of RPGRIP1L, whereas changes in other desmosomal components are secondary or compensatory. Indeed, several prior studies demonstrated the protective role of plakophilin in pemphigus or skin fragility models [34,35,65,66].

In conclusion, data obtained from this study implicate RPGRIP1L in the maintenance of desmosomal junctions through restricting desmoglein endocytosis, thereby revealing a cilia-independent function of RPGRIP1L in epidermal keratinocytes.

## Materials and Methods

### Ethics statement

All procedures related to mice were performed in accordance with the European Directive 2010/63/EU and the French application decree 2013–118 on the protection of animals used for scientific purposes, and were approved by the local ethical committee "Comité d'éthique Charles Darwin" (approval number 2015052909185846), or in accordance with the Guide for the Care and Use of Laboratory Animals of the National Institutes of Health of the United States, and approved by the Institutional Animal Care and Use Committee of Stony Brook University (approval number 2012-1974-R2-9.14.18-MI).

### Mouse model, cell culture, and transfection

The *Rpgrip1l* mouse model was described previously [2,4,5,25]. HaCaT cells were transfected with *RPGRIP1L* siRNAs (HSC.RNAI.N015272.12.1 and 2, Integrated DNA Technologies, Coralville, IA). Non-targeting (Negative Control) siRNA (NC-1, Integrated DNA Technologies) was used as control siRNA. Twenty-four hours after transfection, cells were switched to high calcium (1.5 mM CaCl_2_) for designated durations. EGTA was used at 2 mM. Dynasore (Sigma-Aldrich, Saint Louis, MO) and sucrose were used at 50 μM and 400 mM, respectively. PV IgG were purified in Dr. Payne’s laboratory and used at 400 μg/ml. Normal human IgG (Sigma-Aldrich, Saint Louis, MO) was used as control IgG. Method details are provided in the Supplementary Materials and Methods (S1 Text) online.

### Organotypic explant skin culture

Skins excised from E18.5 embryos were cultured as previously described [67]. Briefly, dorsal skins harvested from E18.5 embryos were cultured at the air-liquid interface in DMEM and 10% fetal bovine serum at 37° C and 5% CO_2_. Cultured skins were then fixed in 10% buffered formalin and processed for routine histology analysis.

### Tissue processing, histology, and immunofluorescence labeling and quantification

Freshly isolated tissues were fixed immediately in buffered formalin, embedded in paraffin, and processed for routine hematoxylin and eosin (H&E) staining or other examinations. Most immunofluorescence labeling of tissue specimens and cells was performed on formalin-fixed paraffin-embedded tissue sections as described previously [68,69]. RPGRIP1L, cilia, DSG3, and CTNNA1 were detected on frozen sections of the skin (Supplementary Methods). TUNEL staining was performed with DeadEnd Fluorometric TUNEL System (Promega, Fitchburg, WI). Primary antibodies are listed in S1 Table. AlexaFluor-conjugated secondary antibodies (1:200) were obtained from Life Technologies (Carlsbad, CA). Sections were sealed in mounting medium with or without DAPI (Vector Laboratories, Burlingame, CA). Images were acquired by a Nikon 80*i* fluorescence microscope, fitted with a Nikon (Melville, NY) DS-Qi1Mc camera, or by a Leica (Wetzlar, Germany) SP5C Spectral confocal laser-scanning microscope, and processed with Photoshop 5.5 CS (Adobe System Incorporated, San Jose, CA).

To quantify fluorescence intensity of skin tissues, the mean intensity of randomly selected epidermal regions (2 regions per specimen, n ≥ 3 mice per group, excluding the cornified layer), was measured with the NIS-Element analysis software, as described elsewhere [50].

To obtain the ratio of membrane/cytoplasmic fluorescence intensity, the mean peak pixel values at the two edges of a cell (representing the plasma membrane) and the mean pixel value between the peaks (representing the cytoplasm) were obtained by the Plot Profile tool of the ImageJ software (1.43u, National Institute of Health, Bethesda, MD), and presented as a membrane/cytoplasmic ratio.

### Dispase dissociation assay

The dispase dissociation assay was performed as described previously [70]. Briefly, 24 hours after transfection, confluent cells were incubated in high calcium medium (1.5 mM CaCl_2_) for 24 hours. Subsequently, cells were washed with DPBS and incubated with dispase II (2.4 U/ml in EMEM with 10% FBS and 1.5 mM CaCl_2_, Roche, Indianapolis, IN), for 20 min at 37°C. Detached monolayers were subjected to mechanical challenge by inverting 50 times in 4 ml PBS in a 15-ml Falcon tube. Cell fragments were imaged and counted under a dissecting microscope (Stemi 2000-C, Carl Zeiss, Obserkochen, Germany).

### DSG3 internalization assay

The IgG internalization assay was performed to detect internalized DSG3 as previously described [27,29]. Briefly, HaCaT cells were incubated with a monoclonal antibody (AK23) against the extracellular domain of DSG3 [41], in media containing 1.5 mM calcium for 30 minutes on ice. Cells were then washed and incubated with PV IgG (400 μg/ml) or normal human IgG (400 μg/ml) at 37° C for one hour to induce DSG3 internalization. Subsequently, cells were treated with acid wash solution (100 mM glycine, 20 mM magnesium acetate, 50 mM potassium chloride, pH 2.2) to remove surface-bound DSG3 antibody before fixation. For knockdown studies, cells were transfected with siRNA prior to calcium switch. Images were acquired by Leica SP5C Spectral confocal laser scanning microscope under the same color intensity threshold and analyzed using ImageJ. Quantification was done by counting green fluorescent puncta in randomly sampled microscopic fields with Analyze Particle, which was then normalized by the number of cells so that the net result reflects the average number of puncta (internalized DSG3) within one cell.

### Cell surface protein biotinylation and endocytosis assay

Cell surface protein biotinylation and endocytosis assays were used to measure internalization of cell surface proteins, as described previously [42]. Briefly, cells were incubated with freshly prepared 2 mg/ml Sulfo-NHS-SS-biotin (EZ-Link™Sulfo-NHS-SS-Biotin; Thermo Fisher Scientific, Waltham, MA) for 30 min at 4°C after two washes in ice-cold PBS^2+^ (PBS with 1.5 mM CaCl_2_ and 1.5 mM MgCl_2_) for biotinylation to occur. Cells were then washed and incubated in three changes of quenching solution (100 mM glycine in PBS^2+^), 10 minutes each, on ice. After a PBS^2+^ wash, cells were incubated in the pre-warmed high calcium growth media containing 2 mM EGTA for 30 min to trigger internalization. Stripping control cells were kept at 4°C to block internalization. Subsequently, non-internalized biotin was stripped by washing with cold NT buffer (150 mM NaCl, 1.0 mM EDTA, 0.2% BSA, 20 mM Tris, pH 8.6, and 50 mM Tris(2-Carboxyethyl) phosphine Hydrochloride), and cell lysates were collected in RIPA buffer containing protease inhibitor. Biotinylated proteins were pulled down with streptavidin magnetic beads (Thermo Fisher Scientific) at 4°C overnight, eluted in Laemmli buffer at 95°C, and analyzed by immunoblotting. Rabbit anti-DSG3 (Bio-Rad AbD Serotec, Raleigh, NC) was used to detect biotinylated DSG3. Target proteins were examined in a minimum of three experiments. Results from one representative experiment are shown.

### Statistical analysis

All quantifications are presented as mean ± SD. Student’s t-test was used unless otherwise stated. One-way ANOVA and two-way ANOVA were conducted using the GraphPad software. *P* < 0.05 was considered statistically significant.

Additional Materials and Methods information is provided in the Supplementary Materials and Methods (S1 Text) online.

## Supporting information

S1 Text Supplementary materials and methods. (PDF)

S1 Table Antibodies used in this study. (PDF)

S1 Fig Expression of RPGRIP1L in mouse skin. **(a**–**h)** Immunofluorescence of RPGRIP1L (green), cilia (acetylated α-tubulin, white), and basal body/centriole (γ-tubulin, red) of E18.5 dorsal skin of wild type (*Rpgrip1l*^+/+^, a and e) and homozygous (*Rpgrip1l*^–/–^, i) mutant mice. Nuclei were stained with DAPI (blue). b–d, f–h, j–l are enlargements of the boxed area in a, e, and i, respectively. Scale bar, 10 μm in (a, e, and i), 1 μm in (b–d, f–h, j–l).
(PDF)

S2 Fig TUNEL assay on E18.5 dorsal skin. (**a** and **b**) TUNEL assay on wild type (*Rpgrip1l*^+/+^) and homozygous (*Rpgrip1l*^*–/–*^) mutant mice. (**c** and **d**) *Rpgrip1l*^+/+^ and *Rpgrip1l*^*–/–*^skins treated with DNase I before subjected to TUNEL assay, as positive controls. Sections were counterstained with DAPI (blue) to label nuclei. Asterisks indicate intraepidermal blisters. Scale bar, 50 μm.
(PDF)

S3 Fig Junctional proteins in E18.5 epidermis. Immunofluorescence labeling of desmocollins (DSC1, DSC2/3, green), E-cadherin (CDH1, red), α-catenin (CTNNA1, green), and β-catenin (CTNNB1, green) in back skin of E18.5 control (*Rpgrip1l*^+/+^) and homozygous (*Rpgrip1l*^–/–^) mutants. Dotted lines illustrate epidermal-dermal junction. Asterisks indicate intraepidermal blisters. Scale bar, 20 μm.
(PDF)

S4 Fig Junctional proteins in E18.5 epidermis. Immunofluorescence labeling (red) of desmocollins (DSC1, DSC2/3), E-cadherin (CDH1), and β-catenin (CTNNB1) in back skin of E18.5 control (*Rpgrip1l*^+/+^) and homozygous (*Rpgrip1l*^–/–^) mutants. Keratin 14 (KRT14) is labeled in green; nuclei are stained blue. Scale bar, 20 μm.
(PDF)

S5 Fig Cell viability in *RPGRIP1L*-knockdown HaCaT cells. Cell viability (%) of mock transfection, control (Control siRNA), and *RPGRIP1L*-knockdown (*RPGRIP1L* siRNA1) HaCaT cells. Bars represent values relative mock transfection.
(PDF)

S6 Fig Gene and protein expression in *RPGRIP1L*-knockdown normal human epidermal keratinocytes (NHEKs). (**a**) Confirmation of *RPGRIP1L* knockdown by qRT-PCR. (**b**) RPGRIP1L, desmogleins (DSGs), desmoplakin (DSP), plakophilins (PKPs), plakoglobin (JUP), and E-cadherin (CDH1) protein levels in control (Control siRNA) and *RPGRIP1L*-knockdown (*RPGRIP1L* siRNA). *** *P* < 0.001; Student’s *t*-test.
(PDF)

S7 Fig Relative mRNA levels of desmosomal genes in *RPGRIP1L*-knockdown HaCaT by qRT-PCR. mRNA level in control (Control siRNA) and *RPGRIP1L*-knockdown (*RPGRIP1L* siRNA) HaCaT were normalized by GAPDH. ** *P* < 0.01, *** *P* < 0.001, ns = not statistically significant.
(PDF)

S8 Fig Immunofluorescence labeling of keratin 14 (KRT14) in control (Control siRNA) and *RPGRIP1L*-knockdown (*RPGRIP1L* siRNA) HaCaT cells. Scale bar, 10 μm.
(PDF)

## References

[1] Yang TT, Su J, Wang WJ, Craige B, Witman GB, et al (2015) Superresolution Pattern Recognition Reveals the Architectural Map of the Ciliary Transition Zone. Sci Rep 5: 14096 10.1038/srep14096 26365165 PMC4568515

[2] Vierkotten J, Dildrop R, Peters T, Wang B, Ruther U (2007) Ftm is a novel basal body protein of cilia involved in Shh signalling. Development 134: 2569–2577. 10.1242/dev.003715 17553904

[3] Mahuzier A, Gaude HM, Grampa V, Anselme I, Silbermann F, et al (2012) Dishevelled stabilization by the ciliopathy protein Rpgrip1l is essential for planar cell polarity. J Cell Biol 198: 927–940. 10.1083/jcb.201111009 22927466 PMC3432770

[4] Besse L, Neti M, Anselme I, Gerhardt C, Ruther U, et al (2011) Primary cilia control telencephalic patterning and morphogenesis via Gli3 proteolytic processing. Development 138: 2079–2088. 10.1242/dev.059808 21490064

[5] Laclef C, Anselme I, Besse L, Catala M, Palmyre A, et al (2015) The role of primary cilia in corpus callosum formation is mediated by production of the Gli3 repressor. Hum Mol Genet 24: 4997–5014. 10.1093/hmg/ddv221 26071364

[6] Delous M, Baala L, Salomon R, Laclef C, Vierkotten J, et al (2007) The ciliary gene RPGRIP1L is mutated in cerebello-oculo-renal syndrome (Joubert syndrome type B) and Meckel syndrome. Nat Genet 39: 875–881. 10.1038/ng2039 17558409

[7] Arts HH, Doherty D, van Beersum SE, Parisi MA, Letteboer SJ, et al (2007) Mutations in the gene encoding the basal body protein RPGRIP1L, a nephrocystin-4 interactor, cause Joubert syndrome. Nat Genet 39: 882–888. 10.1038/ng2069 17558407

[8] Hildebrandt F, Benzing T, Katsanis N (2011) Ciliopathies. N Engl J Med 364: 1533–1543. 10.1056/NEJMra1010172 21506742 PMC3640822

[9] Wiegering A, Ruther U, Gerhardt C (2018) The ciliary protein Rpgrip1l in development and disease. Dev Biol 442: 60–68. 10.1016/j.ydbio.2018.07.024 30075108

[10] Coene KL, Mans DA, Boldt K, Gloeckner CJ, van Reeuwijk J, et al (2011) The ciliopathy-associated protein homologs RPGRIP1 and RPGRIP1L are linked to cilium integrity through interaction with Nek4 serine/threonine kinase. Hum Mol Genet 20: 3592–3605. 10.1093/hmg/ddr280 21685204

[11] Chen J, Laclef C, Moncayo A, Snedecor ER, Yang N, et al (2015) The ciliopathy gene Rpgrip1l is essential for hair follicle development. J Invest Dermatol 135: 701–709. 10.1038/jid.2014.483 25398052 PMC4340706

[12] Elofsson R, Andersson A, Falck B, Sjoborg S (1984) The ciliated human keratinocyte. J Ultrastruct Res 87: 212–220. 6085808 10.1016/s0022-5320(84)80061-1

[13] Nekrasova O, Green KJ (2013) Desmosome assembly and dynamics. Trends Cell Biol 23: 537–546. 10.1016/j.tcb.2013.06.004 23891292 PMC3913269

[14] Holthofer B, Windoffer R, Troyanovsky S, Leube RE (2007) Structure and function of desmosomes. Int Rev Cytol 264: 65–163. 10.1016/S0074-7696(07)64003-0 17964922

[15] Petrof G, Mellerio JE, McGrath JA (2012) Desmosomal genodermatoses. Br J Dermatol 166: 36–45. 10.1111/j.1365-2133.2011.10640.x 21929534

[16] Thomason HA, Scothern A, McHarg S, Garrod DR (2010) Desmosomes: adhesive strength and signalling in health and disease. Biochem J 429: 419–433. 10.1042/BJ20100567 20626351

[17] Bazzi H, Christiano AM (2007) Broken hearts, woolly hair, and tattered skin: when desmosomal adhesion goes awry. Curr Opin Cell Biol 19: 515–520. 10.1016/j.ceb.2007.08.001 17951043 PMC4296313

[18] Amagai M, Stanley JR (2012) Desmoglein as a target in skin disease and beyond. J Invest Dermatol 132: 776–784. 10.1038/jid.2011.390 22189787 PMC3279627

[19] Samuelov L, Sprecher E (2015) Inherited desmosomal disorders. Cell Tissue Res 360: 457–475. 10.1007/s00441-014-2062-y 25487406

[20] Stahley SN, Kowalczyk AP (2015) Desmosomes in acquired disease. Cell Tissue Res 360: 439–456. 10.1007/s00441-015-2155-2 25795143 PMC4456195

[21] Ganeshan R, Chen J, Koch PJ (2010) Mouse models for blistering skin disorders. Dermatol Res Pract 2010: 584353 10.1155/2010/584353 20585602 PMC2879910

[22] O'Shea C, Fitzpatrick JE, Koch PJ (2014) Desmosomal defects in acantholytic squamous cell carcinomas. J Cutan Pathol 41: 873–879. 10.1111/cup.12390 25264142 PMC4245396

[23] Ezratty EJ, Stokes N, Chai S, Shah AS, Williams SE, et al (2011) A role for the primary cilium in Notch signaling and epidermal differentiation during skin development. Cell 145: 1129–1141. 10.1016/j.cell.2011.05.030 21703454 PMC3135909

[24] Wilson RB, McWhorter CA (1963) Isolated flagella in human skin. Election microscopic observations. Lab Invest 12: 242–249. 14001080

[25] Gerhardt C, Lier JM, Kuschel S, Ruther U (2013) The ciliary protein Ftm is required for ventricular wall and septal development. PLoS One 8: e57545 10.1371/journal.pone.0057545 23469020 PMC3585374

[26] Gerhardt C, Lier JM, Burmuhl S, Struchtrup A, Deutschmann K, et al (2015) The transition zone protein Rpgrip1l regulates proteasomal activity at the primary cilium. J Cell Biol 210: 115–133. 10.1083/jcb.201408060 26150391 PMC4494006

[27] Calkins CC, Setzer SV, Jennings JM, Summers S, Tsunoda K, et al (2006) Desmoglein endocytosis and desmosome disassembly are coordinated responses to pemphigus autoantibodies. J Biol Chem 281: 7623–7634. 10.1074/jbc.M512447200 16377623

[28] Jennings JM, Tucker DK, Kottke MD, Saito M, Delva E, et al (2011) Desmosome disassembly in response to pemphigus vulgaris IgG occurs in distinct phases and can be reversed by expression of exogenous Dsg3. J Invest Dermatol 131: 706–718. 10.1038/jid.2010.389 21160493 PMC3235416

[29] Delva E, Jennings JM, Calkins CC, Kottke MD, Faundez V, et al (2008) Pemphigus vulgaris IgG-induced desmoglein-3 endocytosis and desmosomal disassembly are mediated by a clathrin- and dynamin-independent mechanism. J Biol Chem 283: 18303–18313. 10.1074/jbc.M710046200 18434319 PMC2440613

[30] Jolly PS, Berkowitz P, Bektas M, Lee HE, Chua M, et al (2010) p38MAPK signaling and desmoglein-3 internalization are linked events in pemphigus acantholysis. J Biol Chem 285: 8936–8941. 10.1074/jbc.M109.087999 20093368 PMC2838315

[31] Klessner JL, Desai BV, Amargo EV, Getsios S, Green KJ (2009) EGFR and ADAMs cooperate to regulate shedding and endocytic trafficking of the desmosomal cadherin desmoglein 2. Mol Biol Cell 20: 328–337. 10.1091/mbc.E08-04-0356 18987342 PMC2613100

[32] Saito M, Stahley SN, Caughman CY, Mao X, Tucker DK, et al (2012) Signaling dependent and independent mechanisms in pemphigus vulgaris blister formation. PLoS One 7: e50696 10.1371/journal.pone.0050696 23226536 PMC3513318

[33] Vielmuth F, Wanuske MT, Radeva MY, Hiermaier M, Kugelmann D, et al (2018) Keratins Regulate the Adhesive Properties of Desmosomal Cadherins through Signaling. J Invest Dermatol 138: 121–131. 10.1016/j.jid.2017.08.033 28899688

[34] Rietscher K, Wolf A, Hause G, Rother A, Keil R, et al (2016) Growth Retardation, Loss of Desmosomal Adhesion, and Impaired Tight Junction Function Identify a Unique Role of Plakophilin 1 In Vivo. J Invest Dermatol 136: 1471–1478. 10.1016/j.jid.2016.03.021 27033150

[35] Keil R, Rietscher K, Hatzfeld M (2016) Antagonistic Regulation of Intercellular Cohesion by Plakophilins 1 and 3. J Invest Dermatol 136: 2022–2029. 10.1016/j.jid.2016.05.124 27375112

[36] Brennan D, Peltonen S, Dowling A, Medhat W, Green KJ, et al (2012) A role for caveolin-1 in desmoglein binding and desmosome dynamics. Oncogene 31: 1636–1648. 10.1038/onc.2011.346 21841821 PMC3228894

[37] Hobbs RP, Green KJ (2012) Desmoplakin regulates desmosome hyperadhesion. J Invest Dermatol 132: 482–485. 10.1038/jid.2011.318 21993560 PMC3461275

[38] Kroger C, Loschke F, Schwarz N, Windoffer R, Leube RE, et al (2013) Keratins control intercellular adhesion involving PKC-alpha-mediated desmoplakin phosphorylation. J Cell Biol 201: 681–692. 10.1083/jcb.201208162 23690176 PMC3664716

[39] Macia E, Ehrlich M, Massol R, Boucrot E, Brunner C, et al (2006) Dynasore, a cell-permeable inhibitor of dynamin. Dev Cell 10: 839–850. 10.1016/j.devcel.2006.04.002 16740485

[40] Heuser JE, Anderson RG (1989) Hypertonic media inhibit receptor-mediated endocytosis by blocking clathrin-coated pit formation. J Cell Biol 108: 389–400. 2563728 10.1083/jcb.108.2.389 PMC2115439

[41] Tsunoda K, Ota T, Aoki M, Yamada T, Nagai T, et al (2003) Induction of pemphigus phenotype by a mouse monoclonal antibody against the amino-terminal adhesive interface of desmoglein 3. J Immunol 170: 2170–2178. 12574390 10.4049/jimmunol.170.4.2170

[42] Gabriel L, Stevens Z, Melikian H (2009) Measuring plasma membrane protein endocytic rates by reversible biotinylation. J Vis Exp 34: 1669. 10.3791/1669 PMC3152245 20032927

[43] Overmiller AM, McGuinn KP, Roberts BJ, Cooper F, Brennan-Crispi DM, et al (2016) c-Src/Cav1-dependent activation of the EGFR by Dsg2. Oncotarget 7: 37536–37555. 10.18632/oncotarget.7675 26918609 PMC5122330

[44] Bektas M, Jolly PS, Berkowitz P, Amagai M, Rubenstein DS (2013) A pathophysiologic role for epidermal growth factor receptor in pemphigus acantholysis. J Biol Chem 288: 9447–9456. 10.1074/jbc.M112.438010 23404504 PMC3611014

[45] Moftah H, Dias K, Apu EH, Liu L, Uttagomol J, et al (2017) Desmoglein 3 regulates membrane trafficking of cadherins, an implication in cell-cell adhesion. Cell Adh Migr 11: 211–232. 10.1080/19336918.2016.1195942 27254775 PMC5479455

[46] Rotzer V, Hartlieb E, Vielmuth F, Gliem M, Spindler V, et al (2015) E-cadherin and Src associate with extradesmosomal Dsg3 and modulate desmosome assembly and adhesion. Cell Mol Life Sci 72: 4885–4897. 10.1007/s00018-015-1977-0 26115704 PMC11113844

[47] Cirillo N, Prime SS (2009) Desmosomal interactome in keratinocytes: a systems biology approach leading to an understanding of the pathogenesis of skin disease. Cell Mol Life Sci 66: 3517–3533. 10.1007/s00018-009-0139-7 19756386 PMC11115514

[48] Baldari CT, Rosenbaum J (2010) Intraflagellar transport: it's not just for cilia anymore. Curr Opin Cell Biol 22: 75–80. 10.1016/j.ceb.2009.10.010 19962875 PMC3789623

[49] Sang L, Miller JJ, Corbit KC, Giles RH, Brauer MJ, et al (2011) Mapping the NPHP-JBTS-MKS protein network reveals ciliopathy disease genes and pathways. Cell 145: 513–528. 10.1016/j.cell.2011.04.019 21565611 PMC3383065

[50] Sumigray KD, Chen H, Lechler T (2011) Lis1 is essential for cortical microtubule organization and desmosome stability in the epidermis. J Cell Biol 194: 631–642. 10.1083/jcb.201104009 21844209 PMC3160577

[51] Rotzer V, Breit A, Waschke J, Spindler V (2014) Adducin is required for desmosomal cohesion in keratinocytes. J Biol Chem 289: 14925–14940. 10.1074/jbc.M113.527127 24711455 PMC4031542

[52] Vollner F, Ali J, Kurrle N, Exner Y, Eming R, et al (2016) Loss of flotillin expression results in weakened desmosomal adhesion and Pemphigus vulgaris-like localisation of desmoglein-3 in human keratinocytes. Sci Rep 6: 28820 10.1038/srep28820 27346727 PMC4922016

[53] Bass-Zubek AE, Hobbs RP, Amargo EV, Garcia NJ, Hsieh SN, et al (2008) Plakophilin 2: a critical scaffold for PKC alpha that regulates intercellular junction assembly. J Cell Biol 181: 605–613. 10.1083/jcb.200712133 18474624 PMC2386101

[54] Godsel LM, Hsieh SN, Amargo EV, Bass AE, Pascoe-McGillicuddy LT, et al (2005) Desmoplakin assembly dynamics in four dimensions: multiple phases differentially regulated by intermediate filaments and actin. J Cell Biol 171: 1045–1059. 10.1083/jcb.200510038 16365169 PMC2171300

[55] Dehner C, Rotzer V, Waschke J, Spindler V (2014) A desmoplakin point mutation with enhanced keratin association ameliorates pemphigus vulgaris autoantibody-mediated loss of cell cohesion. Am J Pathol 184: 2528–2536. 10.1016/j.ajpath.2014.05.016 25010392

[56] Kimura TE, Merritt AJ, Lock FR, Eckert JJ, Fleming TP, et al (2012) Desmosomal adhesiveness is developmentally regulated in the mouse embryo and modulated during trophectoderm migration. Dev Biol 369: 286–297. 10.1016/j.ydbio.2012.06.025 22819675

[57] Spindler V, Dehner C, Hubner S, Waschke J (2014) Plakoglobin but not desmoplakin regulates keratinocyte cohesion via modulation of p38MAPK signaling. J Invest Dermatol 134: 1655–1664. 10.1038/jid.2014.21 24441103

[58] Mao X, Choi EJ, Payne AS (2009) Disruption of desmosome assembly by monovalent human pemphigus vulgaris monoclonal antibodies. J Invest Dermatol 129: 908–918. 10.1038/jid.2008.339 19037235 PMC2743719

[59] Mao X, Sano Y, Park JM, Payne AS (2011) p38 MAPK activation is downstream of the loss of intercellular adhesion in pemphigus vulgaris. J Biol Chem 286: 1283–1291. 10.1074/jbc.M110.172874 21078676 PMC3020736

[60] Roepman R, Letteboer SJ, Arts HH, van Beersum SE, Lu X, et al (2005) Interaction of nephrocystin-4 and RPGRIP1 is disrupted by nephronophthisis or Leber congenital amaurosis-associated mutations. Proc Natl Acad Sci U S A 102: 18520–18525. 10.1073/pnas.0505774102 16339905 PMC1317916

[61] Delous M, Hellman NE, Gaude HM, Silbermann F, Le Bivic A, et al (2009) Nephrocystin-1 and nephrocystin-4 are required for epithelial morphogenesis and associate with PALS1/PATJ and Par6. Hum Mol Genet 18: 4711–4723. 10.1093/hmg/ddp434 19755384 PMC2778369

[62] Rubsam M, Broussard JA, Wickstrom SA, Nekrasova O, Green KJ, et al (2018) Adherens Junctions and Desmosomes Coordinate Mechanics and Signaling to Orchestrate Tissue Morphogenesis and Function: An Evolutionary Perspective. Cold Spring Harb Perspect Biol 10: a029207 10.1101/cshperspect.a029207 28893859 PMC6211388

[63] Shafraz O, Rubsam M, Stahley SN, Caldara AL, Kowalczyk AP, et al (2018) E-cadherin binds to desmoglein to facilitate desmosome assembly. Elife 7: e37629 10.7554/eLife.37629 29999492 PMC6066328

[64] Struchtrup A, Wiegering A, Stork B, Ruther U, Gerhardt C (2018) The ciliary protein RPGRIP1L governs autophagy independently of its proteasome-regulating function at the ciliary base in mouse embryonic fibroblasts. Autophagy 14: 567–583. 10.1080/15548627.2018.1429874 29372668 PMC5959336

[65] Tucker DK, Stahley SN, Kowalczyk AP (2014) Plakophilin-1 protects keratinocytes from pemphigus vulgaris IgG by forming calcium-independent desmosomes. J Invest Dermatol 134: 1033–1043. 10.1038/jid.2013.401 24056861 PMC3961504

[66] South AP, Wan H, Stone MG, Dopping-Hepenstal PJ, Purkis PE, et al (2003) Lack of plakophilin 1 increases keratinocyte migration and reduces desmosome stability. J Cell Sci 116: 3303–3314. 10.1242/jcs.00636 12840072

[67] Zhang Y, Tomann P, Andl T, Gallant NM, Huelsken J, et al (2009) Reciprocal requirements for EDA/EDAR/NF-kappaB and Wnt/beta-catenin signaling pathways in hair follicle induction. Dev Cell 17: 49–61. 10.1016/j.devcel.2009.05.011 19619491 PMC2859042

[68] Dai D, Li L, Huebner A, Zeng H, Guevara E, et al (2013) Planar cell polarity effector gene Intu regulates cell fate-specific differentiation of keratinocytes through the primary cilia. Cell Death Differ 20: 130–138. 10.1038/cdd.2012.104 22935613 PMC3524640

[69] Yang N, Li L, Eguether T, Sundberg JP, Pazour GJ, et al (2015) Intraflagellar transport 27 is essential for hedgehog signaling but dispensable for ciliogenesis during hair follicle morphogenesis. Development 142: 2194–2202. 10.1242/dev.115261 26023097 PMC4483764

[70] Hobbs RP, Amargo EV, Somasundaram A, Simpson CL, Prakriya M, et al (2011) The calcium ATPase SERCA2 regulates desmoplakin dynamics and intercellular adhesive strength through modulation of PKCalpha signaling. FASEB J 25: 990–1001. 10.1096/fj.10-163261 21156808 PMC3042836

