# Supplementary material for: RPGRIP1L is required for stabilizing epidermal keratinocyte adhesion through regulating desmoglein endocytosis
Source: PLoS Genet. 2019 Jan 28;15(1):e1007914. doi: 10.1371/journal.pgen.1007914 (PMC6366717; doi:10.1371/journal.pgen.1007914)
Supplement: S1 Text — (PDF) [file pgen.1007914.s001.pdf]

SUPPLEMENTARY MATERIALS AND METHODS

Cell culture and transfection

HaCaT cells were cultured in Eagle's Minimum Essential Medium (Lonza, Walkersville, MD) supplemented with 10% fetal bovine serum (Mediatech, Manassas, VA), 50 U/ml penicillin and 50 µg/ml streptomycin (Lonza, Walkersville, MD) and maintained in a humidified atmosphere containing 5 % CO_2 at 37 °C. For knockdown experiments, siRNAs were transfected with RNAiMAX™ (Life Technologies, Carlsbad, CA) to 3 x10^5 cells/well in 6-well plates following the manufacturer's recommendations. Mock transfection without siRNA was performed to control for transfection associated toxicity or nonspecific effect. Control siRNA (NC1) and three *RPGRIP1L* siRNA (HSC.RNAI.N015272.12.1 - 12.3) are purchased from Integrated DNA Technologies (Coralville, IA) and 12.1 and 12.2 were used. Most of data obtained from HSC.RNAI.N015272.12.1 were shown. Twenty-four hours after transfection, cells were switched to high calcium (1.5 mM $CaCl_2$) for designated durations. EGTA were used at 2 mM. Dynasore (Sigma-Aldrich, Saint Louis, MO) and sucrose were used at 50 μM and 400 mM, respectively. PV IgG were purified in Dr. Payne's laboratory and used at 400 μg/ml. Normal human IgG (Sigma-Aldrich, Saint Louis, MO) were used as control IgG.

Cell viability assay

HaCaT cells were transfected with siRNA for 24 or 48 hours in 96-well plates. Thereafter, 10 μl of alamarBlue™ cell viability assay reagent (Thermo Scientific, Wilmington, DE) was added and incubated at 37 °C for 2 hours. Absorbance at a wavelength of 570 nm and 600 nm was measured. The reduction of alamarBlue reagent was calculated following manufacturer's instruction. Cell viability was shown in relative to mock transfection.

Immunofluorescence labeling of RPGRIP1L, cilia, DSG3, and CTNNA1 in skin

Immunofluorescence staining of RPGRIP1L, cilia, and basal bodies was performed on 14 μm cryosections, as described previously [1,2]. After rehydration, sections were subject to antigen retrieval by boiling for 7 min with 10 mM citrate buffer, pH 6.0, and incubated with primary and secondary antibodies. Nuclei were stained with DAPI. Images were taken with a Leica TCS SP5 AOBS confocal microscope. DSG3 and CTNNA1 were detected on 5 μm frozen sections. DSG3 immunofluorescence was performed as described previously [3]. Briefly, the DSG3 antibody, PV4B3 scFv (1 mg/mL, dilute 1:100) purified in the Payne laboratory was used. Binding was detected by staining with rat anti-HA mAb (3F10; dilution, 1:100; Roche Diagnostics), followed by an Alexa Flour 488-conjugated anti-rat IgG (dilution, 1:200; Invitrogen).

In situ hybridization

In situ hybridization was performed with the RNAScope *in situ* hybridization as described previously [4].

RNA isolation and quantitative RT-PCR

RNA was isolated with the RNeasy kit (Qiagen) and quantitative RT-PCR analyses were performed as described previously [5]. Briefly, complementary DNA was synthesized from 1 µg of total mRNA using a SuperScript II First-Strand Synthesis System (Invitrogen, Carlsbad, CA) and random hexameric primers. Real-time qRT-PCR was performed on ABI Prism 7500 (Applied Biosystems, Foster City, CA) with the following TaqMan probes (Life Technologies, Grand Island, NY): RPGRIP1L, Hs00324332_m1; DSG1, Hs00355084_m1; DSG2, Hs00170071_m1; DSG3, Hs00951897_m1; DSP, Hs00950591_m1; PKP1, Hs00240873_m1; PKP2, Hs00428040_m1; PKP3, Hs00170887_m1; GAPDH, Hs02758991_g1. Results were analyzed using ΔΔCt method. Relative expression levels of target genes were determined by comparing with wild type or treatment controls after normalizing with GAPDH.

Protein Analysis

Protein was extracted either by homogenizing tissue or cells in cold RIPA lysis buffer (50 mM Tris-HCl pH 7.4, 150 mM NaCl, 1% Triton X-100, 1% sodium deoxycholate, and 0.1% SDS) supplemented with proteinase inhibitors. Cells lysates were cleared by centrifugation at 13,000 × g for 20 min at 4 ºC; protein concentration was determined by BCA Protein assay Kit (Pierce) or NanoDrop 2000 spectrophotometer (Thermo Scientific, Wilmington, DE). Equal amounts of protein were loaded on a 10% SDS-PAGE and transferred to Hybond Nitrocellulose (GE Healthcare) membranes and probed with the primary antibodies at 4 °C. Blots were then incubated with HRP-labeled secondary antibody for 1 hour. SuperSignal substrates (Thermo Scientific) and CL-XPosure film (Thermo Scientific) were used for detection. Quantification was performed by using densitometry and ImageJ software (National Institute of Health, Bethesda, USA).

TEM

Dorsal skin dissected from E18.5 embryos was fixed in 2% glutaraldehyde, 4% PFA, 1 mM $CaCl_2$, and 0.05 M cacodylate, pH 7.4, for 1 hour at room temperature and then overnight at 4°C. After fixation, samples were placed in 2% osmium tetroxide in 0.1 M sodium phosphate buffer (pH 7.4), dehydrated in graded ethyl alcohol and embedded in Embed812 resin. Ultrathin sections of 80 nm were prepared with a Leica EM UC7 ultramicrotome. For cultured cells, cells were plated on ACLAR sheets and treated with siRNA and high calcium media described above.

Cells were fixed with 2.5% EM grade glutaraldehyde in 0.1 M phosphate buffer (pH 7.4) for 1 hour. Samples were then placed in 1% osmium tetroxide in 0.1 M PBS (pH 7.4), dehydrated in graded ethyl alcohol and embedded in Durcupan resin. The ACLAR sheets were peeled away and glued onto a blank resin block. Ultrathin sections of 80 nm were cut with a Reichert-Jung Ultracut E ultramicrotome. Sections were counterstained with uranyl acetate and lead citrate and viewed with a FEI Tecnai12 BioTwinG2 electron microscope. Digital images were acquired with an AMT XR-60 CCD digital camera and processed with Photoshop 5.5 CS.

REFERENCES

1. Anselme I, Laclef C, Lanaud M, Ruther U, Schneider-Maunoury S (2007) Defects in brain patterning and head morphogenesis in the mouse mutant Fused toes. Dev Biol 304: 208-220.
2. Laclef C, Anselme I, Besse L, Catala M, Palmyre A, et al. (2015) The role of primary cilia in corpus callosum formation is mediated by production of the Gli3 repressor. Hum Mol Genet 24: 4997-5014.
3. Payne AS, Ishii K, Kacir S, Lin C, Li H, et al. (2005) Genetic and functional characterization of human pemphigus vulgaris monoclonal autoantibodies isolated by phage display. J Clin Invest 115: 888-899.
4. Chen J, Laclef C, Moncayo A, Snedecor ER, Yang N, et al. (2015) The ciliopathy gene Rpgrip1l is essential for hair follicle development. J Invest Dermatol 135: 701-709.
5. Dai D, Li L, Huebner A, Zeng H, Guevara E, et al. (2013) Planar cell polarity effector gene Intu regulates cell fate-specific differentiation of keratinocytes through the primary cilia. Cell Death Differ 20: 130-138.
