# Supplementary material for: RPGRIP1L is required for stabilizing epidermal keratinocyte adhesion through regulating desmoglein endocytosis
Source: PLoS Genet. 2019 Jan 28;15(1):e1007914. doi: 10.1371/journal.pgen.1007914 (PMC6366717; doi:10.1371/journal.pgen.1007914)
Supplement: S1 Table — (PDF) [file pgen.1007914.s002.pdf]

S1 Table

Primary antibody	Supplier	Host Species	Dilution
β-Actin	Santa Cruz Biotechnology, Santa Cruz, CA	Mouse	1:2000 (WB)
ARL13B	NeuroMab, Davis, CA	Mouse	1:100 (IF)
E-Cadherin	BD Bioscience, San Jose, CA	Mouse	1:500 (IF), 1:2000 (WB)
α-Catenin	Sigma-Aldrich, Saint Louis, MO	Rabbit	1:2500 (IF)
β-Catenin	BD Bioscience, San Jose, CA	Mouse	1:200 (IF)
DSC1	Santa Cruz Biotechnology, Santa Cruz, CA	Mouse	1:100 (IF)
DSC2/3	Santa Cruz Biotechnology, Santa Cruz, CA	Mouse	1:100 (IF), 1:500 (WB)
DSC3	Meridian Life Science Inc, Memphis, TN	Mouse	1:25 (IF), 1:200 (WB)
DSG1/2	Progen, Heidelberg, Germany	Mouse	1:80 (IF in cell), 1:10 (IF in skin), 1:500 (WB)
DSG3	Life Technologies, Waltham, MA	Mouse	1:100 (IF), 1:1000 (WB)
DSG3	Bio-Rad AbD Serotec, Raleigh, NC	Rabbit	1:500 (WB)
DSG3	Payne laboratory (PV4B3 scFv) [§]	Human	1:100 (IF)
DSP1/2	Progen, Heidelberg, Germany	Mouse	Undiluted (IF)
DSP1/2	Green laboratory [¶]	Rabbit	1:3000 (WB)
EGFR	Cell Signaling Technology, Danvers, MA	Rabbit	1:1000 (WB)
JUP	Progen, Heidelberg, Germany	Guinea pig	1:100 (IF), 1:1000 (WB)
KRT1	Roop laboratory [#]	Rabbit	1:500 (IF)
KRT14	BioLegend, San Diego, CA	Chicken	1:1000 (IF)
PKP1	Progen, Heidelberg, Germany	Guinea pig	1:100 (IF), 1:1000 (WB)
PKP2	Progen, Heidelberg, Germany	Mouse	Undiluted (IF), 1:100 (WB)
PKP3	Progen, Heidelberg, Germany	Mouse	Undiluted (IF), 1:500 (WB)
RPGRIP1L	Proteintech Group, Rosemont, IL	Rabbit	1:1000 (WB)
RPGRIP1L	Schneider-Maunoury laboratory	Rabbit	1:100 (IF)
Acetylated α-tubulin	Sigma-Aldrich, Saint Louis, MO	Mouse	1:200 (IF)
γ-Tubulin	Sigma-Aldrich, Saint Louis, MO	Mouse	1:500 (IF)
γ-Tubulin	Abcam, Cambridge, MA	Rabbit	1:500 (IF)

WB, western blotting; IF, immunofluorescence

[#] Roop DR, Huitfeldt H, Kilkenny A, Yuspa SH (1987) Differentiation 35: 143-150.

[§] Payne AS, Ishii K, Kacir S, Lin C, Li H, Hanakawa Y, Tsunoda K, Amagai M, Stanley JR, Siegel DL (2005) J Clin Invest 115: 888-899.

[¶] Bornslaeger EA, Corcoran CM, Stappenbeck TS, Green KJ (1996) J Cell Biol 134: 985-1001.
