# Supplementary material for: RPGRIP1L is required for stabilizing epidermal keratinocyte adhesion through regulating desmoglein endocytosis
Source: PLoS Genet. 2019 Jan 28;15(1):e1007914. doi: 10.1371/journal.pgen.1007914 (PMC6366717; doi:10.1371/journal.pgen.1007914)
Supplement: S1 Fig — (a–h) Immunofluorescence of RPGRIP1L (green), cilia (acetylated α-tubulin, white), and basal body/centriole (γ-tubulin, red) of E18.5 dorsal skin of wild type (Rpgrip1l+/+, a and e) and homozygous (Rpgrip1l–/–, i) mutant mice. Nuclei were stained with DAPI (blue). b–d, f–h, j–l are enlargements of the boxed area in a, e, and i, respectively. Scale bar, 10 μm in (a, e, and i), 1 μm in (b–d, f–h, j–l). (PDF) [file pgen.1007914.s003.pdf]

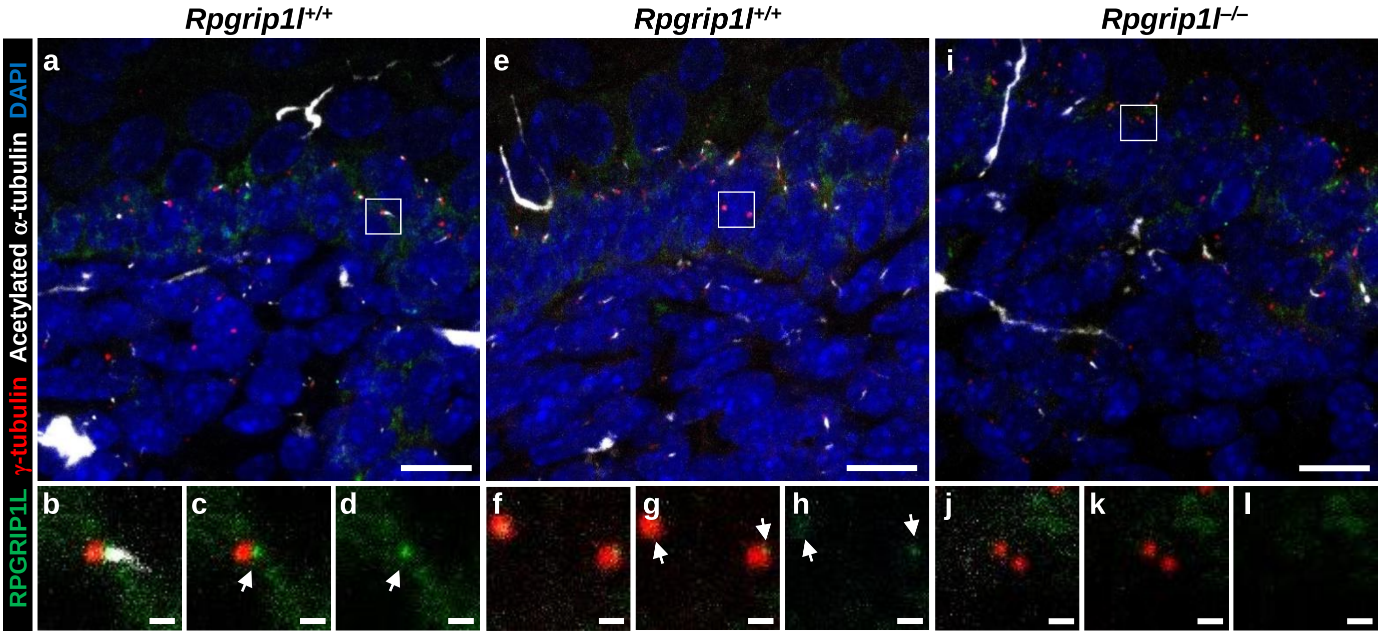


**S1 Fig. Expression of RPGRIP1L in mouse skin. (a – l)** Immunofluorescence of RPGRIP1L (green), cilia (acetylated α-tubulin, white), and basal body/centriole (γ-tubulin, red) on E18.5 dorsal skin of wild type (*Rpgrip1l*$^{+/+}$, a and e) and homozygous (*Rpgrip1l*$^{-/-}$, i) mutant mice. Nuclei were stained with DAPI (blue). b – d, f – h, and j – l are enlarged boxed area in a, e, and i respectively. Scale bar, 10 μm in (a, e, i), 1 μm in (b – d, f – h, j – l).
