# Supplementary material for: RPGRIP1L is required for stabilizing epidermal keratinocyte adhesion through regulating desmoglein endocytosis
Source: PLoS Genet. 2019 Jan 28;15(1):e1007914. doi: 10.1371/journal.pgen.1007914 (PMC6366717; doi:10.1371/journal.pgen.1007914)
Supplement: S2 Fig — (a and b) TUNEL assay on wild type (Rpgrip1l+/+) and homozygous (Rpgrip1l–/–) mutant mice. (c and d) Rpgrip1l+/+ and Rpgrip1l–/–skins treated with DNase I before subjected to TUNEL assay, as positive controls. Sections were counterstained with DAPI (blue) to label nuclei. Asterisks indicate intraepidermal blisters. Scale bar, 50 μm. (PDF) [file pgen.1007914.s004.pdf]

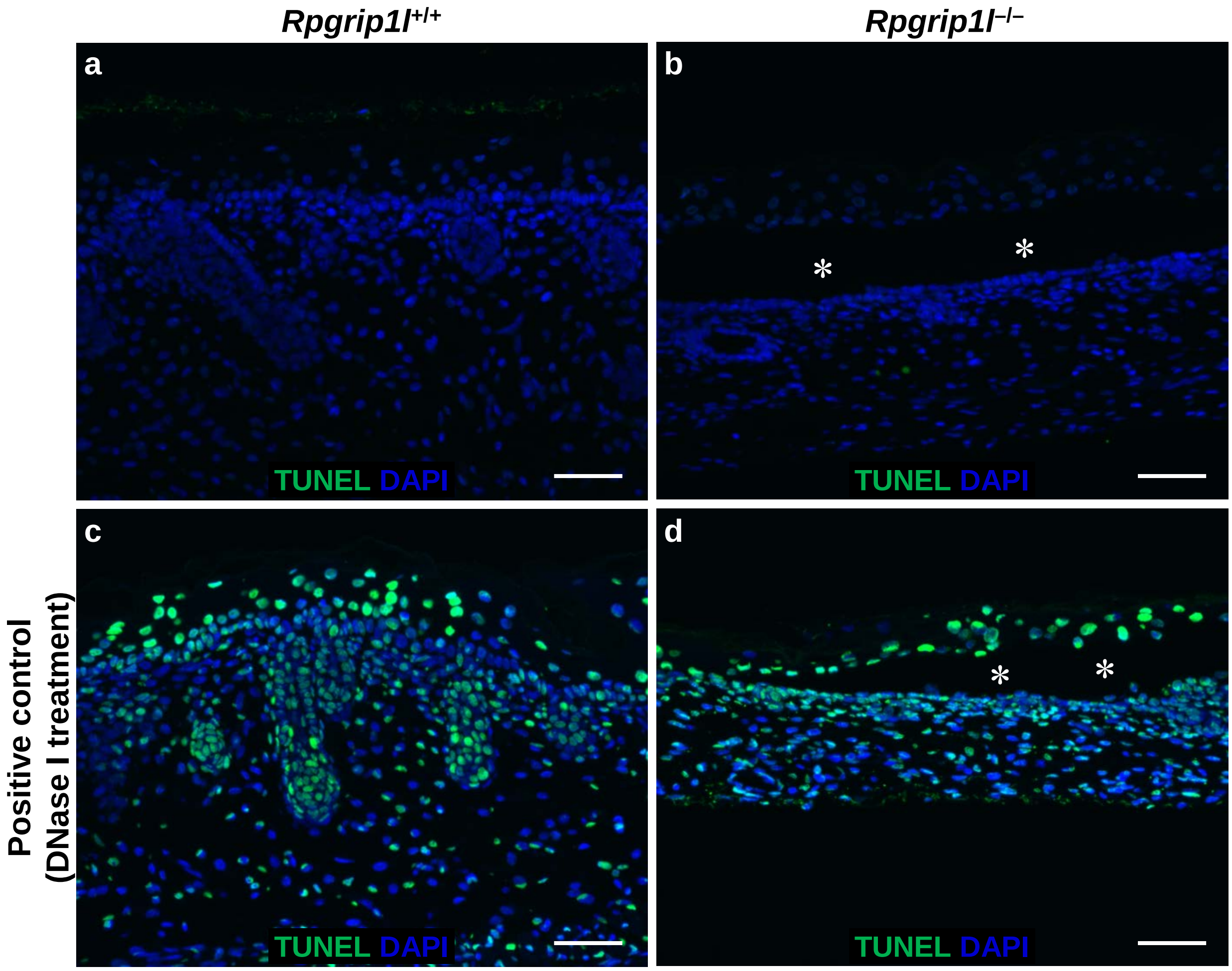

S2 Fig. TUNEL assay on E18.5 dorsal skin. (**a** and **b**) TUNEL assay on wild type (*Rpgrip1l*$^{+/+}$) and homozygous (*Rpgrip1l*$^{-/-}$) mutant mice. (**c** and **d**) *Rpgrip1l*$^{+/+}$ and *Rpgrip1l*$^{-/-}$skins treated with DNase I before subjected to TUNEL assay, as a positive controls. Sections were counterstained with DAPI (blue) to label nuclei. Asterisks indicate intraepidermal blisters. Scale bar, 50 µm.
