# Supplementary material for: RPGRIP1L is required for stabilizing epidermal keratinocyte adhesion through regulating desmoglein endocytosis
Source: PLoS Genet. 2019 Jan 28;15(1):e1007914. doi: 10.1371/journal.pgen.1007914 (PMC6366717; doi:10.1371/journal.pgen.1007914)
Supplement: S3 Fig — Immunofluorescence labeling of desmocollins (DSC1, DSC2/3, green), E-cadherin (CDH1, red), α-catenin (CTNNA1, green), and β-catenin (CTNNB1, green) in back skin of E18.5 control (Rpgrip1l+/+) and homozygous (Rpgrip1l–/–) mutants. Dotted lines illustrate epidermal-dermal junction. Asterisks indicate intraepidermal blisters. Scale bar, 20 μm. (PDF) [file pgen.1007914.s005.pdf]

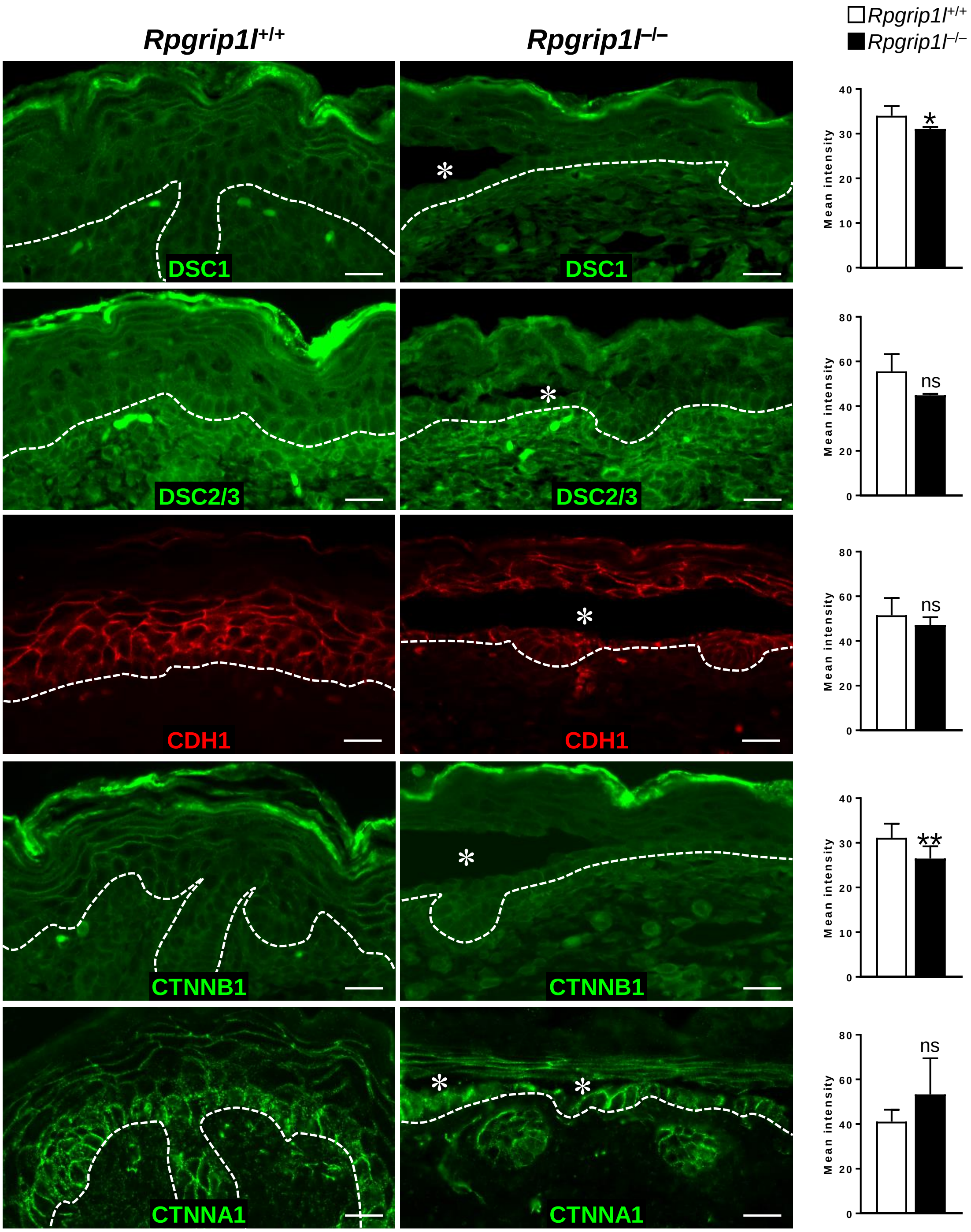


**S3 Fig. Junctional proteins in E18.5 epidermis.** Immunofluorescence labeling of desmocollins (DSC1, DSC2/3, green), E-cadherin (CDH1, red), α-catenin (CTNNA1, green), and β-catenin (CTNNB1, green) in back skin of E18.5 control (*Rpgrip1l*$^{+/+}$) and homozygous (*Rpgrip1l*$^{-/-}$) mutants. Dotted lines illustrate epidermal-dermal junction. Asterisks indicate intraepidermal blisters. Scale bar, 20 µm.
