# Supplementary material for: RPGRIP1L is required for stabilizing epidermal keratinocyte adhesion through regulating desmoglein endocytosis
Source: PLoS Genet. 2019 Jan 28;15(1):e1007914. doi: 10.1371/journal.pgen.1007914 (PMC6366717; doi:10.1371/journal.pgen.1007914)
Supplement: S4 Fig — Immunofluorescence labeling (red) of desmocollins (DSC1, DSC2/3), E-cadherin (CDH1), and β-catenin (CTNNB1) in back skin of E18.5 control (Rpgrip1l+/+) and homozygous (Rpgrip1l–/–) mutants. Keratin 14 (KRT14) is labeled in green; nuclei are stained blue. Scale bar, 20 μm. (PDF) [file pgen.1007914.s006.pdf]

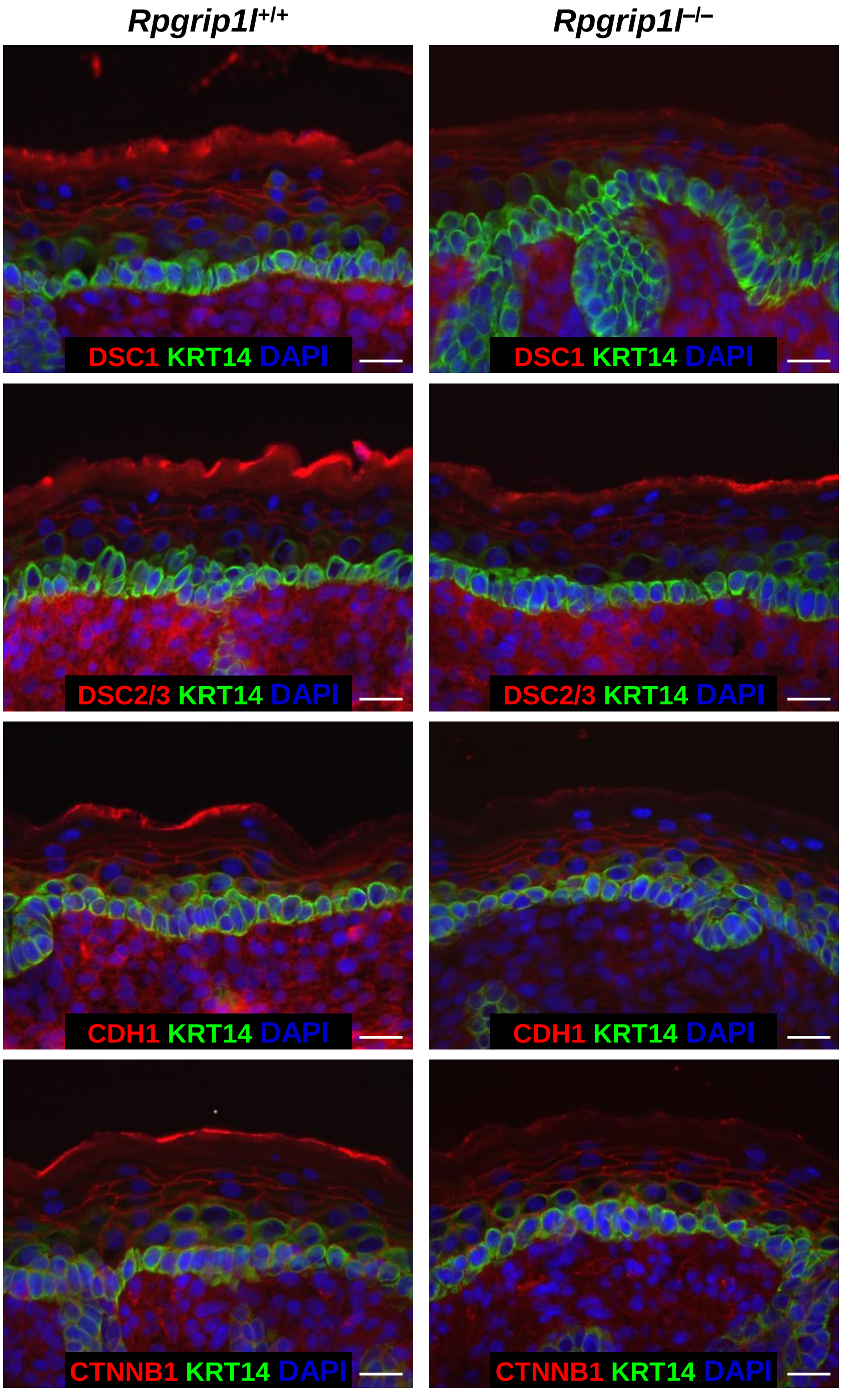

S4 Fig. Junctional proteins in E18.5 epidermis. Immunofluorescence labeling (red) of desmocollins (DSC1, DSC2/3), E-cadherin (CDH1), β-catenin (CTNNB1) in back skin of E18.5 control (*Rpgrip1l*$^{+/+}$) and homozygous (*Rpgrip1l*$^{-/-}$) mutants. Keratin 14 (KRT14) is labeled in green; nuclei were stained blue. Scale bar, 20 µm.
