# Supplementary material for: RPGRIP1L is required for stabilizing epidermal keratinocyte adhesion through regulating desmoglein endocytosis
Source: PLoS Genet. 2019 Jan 28;15(1):e1007914. doi: 10.1371/journal.pgen.1007914 (PMC6366717; doi:10.1371/journal.pgen.1007914)
Supplement: S5 Fig — Cell viability (%) of mock transfection, control (Control siRNA), and RPGRIP1L-knockdown (RPGRIP1L siRNA1) HaCaT cells. Bars represent values relative mock transfection. (PDF) [file pgen.1007914.s007.pdf]

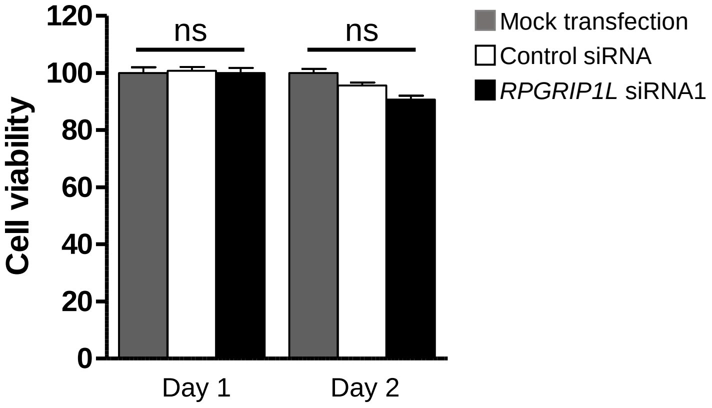

S5 Fig. Cell viability in *RPGRIP1L*-knockdown HaCaT cells. Cell viability (%) of mock transfection, control (Control siRNA) and *RPGRIP1L*-knockdown (*RPGRIP1L* siRNA1) HaCaT cells. Bars represent values relative mock transfection.
