# Supplementary material for: RPGRIP1L is required for stabilizing epidermal keratinocyte adhesion through regulating desmoglein endocytosis
Source: PLoS Genet. 2019 Jan 28;15(1):e1007914. doi: 10.1371/journal.pgen.1007914 (PMC6366717; doi:10.1371/journal.pgen.1007914)
Supplement: S6 Fig — (a) Confirmation of RPGRIP1L knockdown by qRT-PCR. (b) RPGRIP1L, desmogleins (DSGs), desmoplakin (DSP), plakophilins (PKPs), plakoglobin (JUP), and E-cadherin (CDH1) protein levels in control (Control siRNA) and RPGRIP1L-knockdown (RPGRIP1L siRNA). *** P < 0.001; Student’s t-test. (PDF) [file pgen.1007914.s008.pdf]

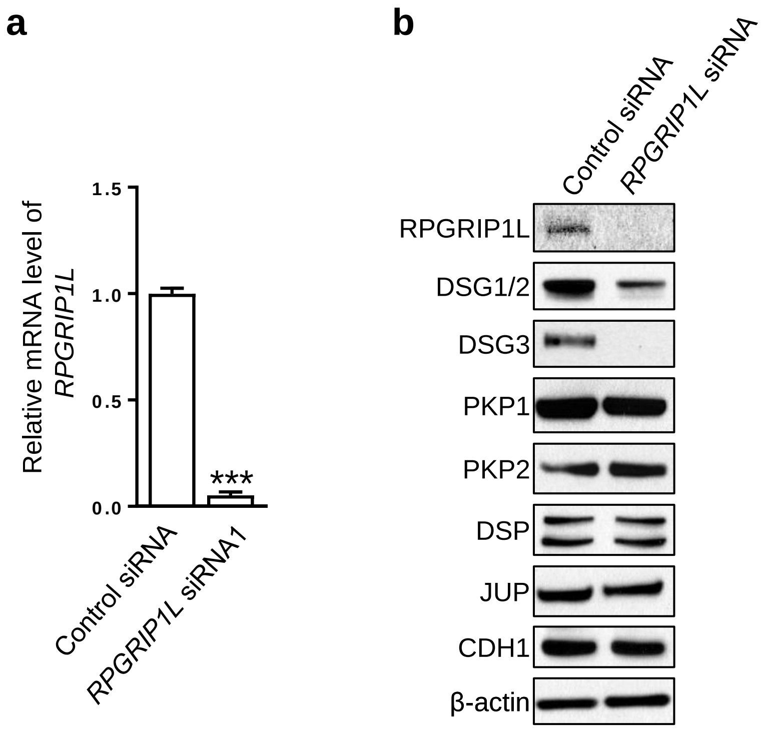

S6 Fig. Gene and protein expression in *RPGRIP1L*-knockdown normal human epidermal keratinocytes (NHEKs). (**a**) Confirmation of *RPGRIP1L* knockdown by qRT-PCR. (**b**) RPGRIP1L, desmogleins (DSGs), desmoplakin (DSP), plakophilins (PKPs), plakoglobin (JUP), and E-cadherin (CDH1) protein levels in control (Control siRNA) and *RPGRIP1L*-knockdown (*RPGRIP1L* siRNA). *** $P < 0.001$; Student's *t*-test.
