# Supplementary material for: RPGRIP1L is required for stabilizing epidermal keratinocyte adhesion through regulating desmoglein endocytosis
Source: PLoS Genet. 2019 Jan 28;15(1):e1007914. doi: 10.1371/journal.pgen.1007914 (PMC6366717; doi:10.1371/journal.pgen.1007914)
Supplement: S7 Fig — mRNA level in control (Control siRNA) and RPGRIP1L-knockdown (RPGRIP1L siRNA) HaCaT were normalized by GAPDH. ** P < 0.01, *** P < 0.001, ns = not statistically significant. (PDF) [file pgen.1007914.s009.pdf]

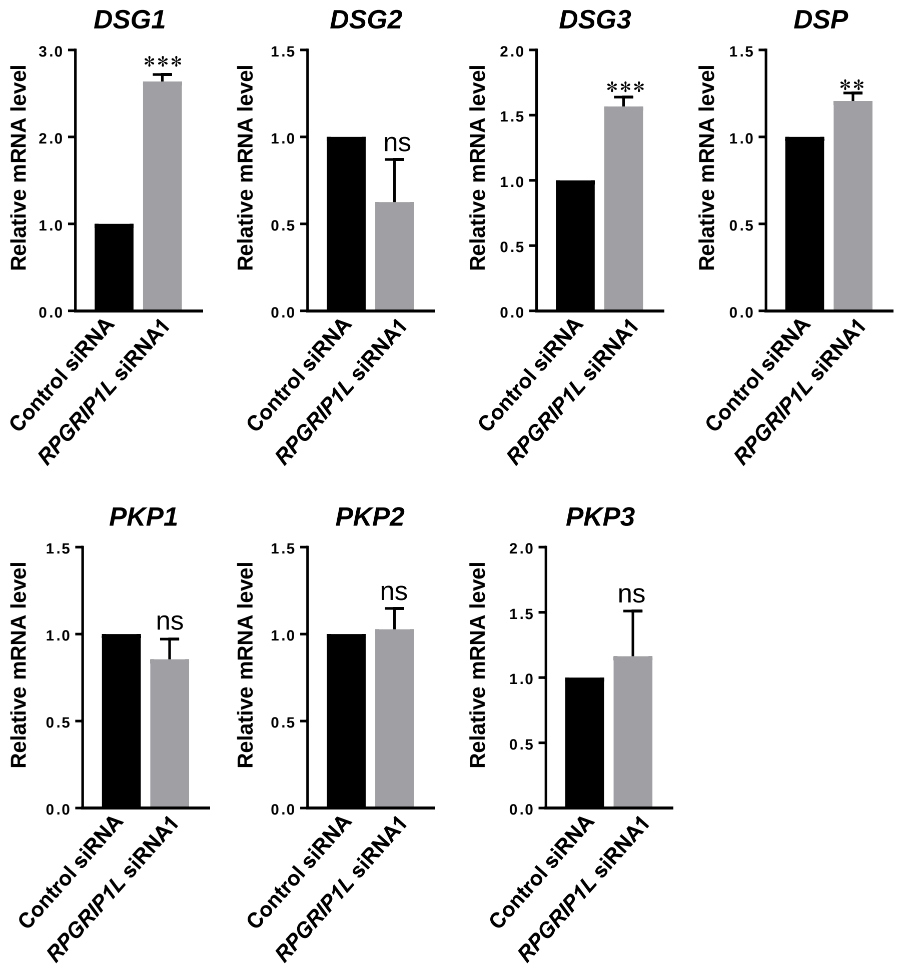


**S7 Fig. Relative mRNA levels of desmosomal genes in *RPGRIP1L*-knockdown HaCaT by qRT-PCR.** mRNA level in control (Control siRNA) and *RPGRIP1L*-knockdown (*RPGRIP1L* siRNA) HaCaT were normalized by GAPDH. ** $P < 0.01$, *** $P < 0.001$, ns = not statistically significant.
