# Supplementary material for: RPGRIP1L is required for stabilizing epidermal keratinocyte adhesion through regulating desmoglein endocytosis
Source: PLoS Genet. 2019 Jan 28;15(1):e1007914. doi: 10.1371/journal.pgen.1007914 (PMC6366717; doi:10.1371/journal.pgen.1007914)
Supplement: S8 Fig — Scale bar, 10 μm. (PDF) [file pgen.1007914.s010.pdf]

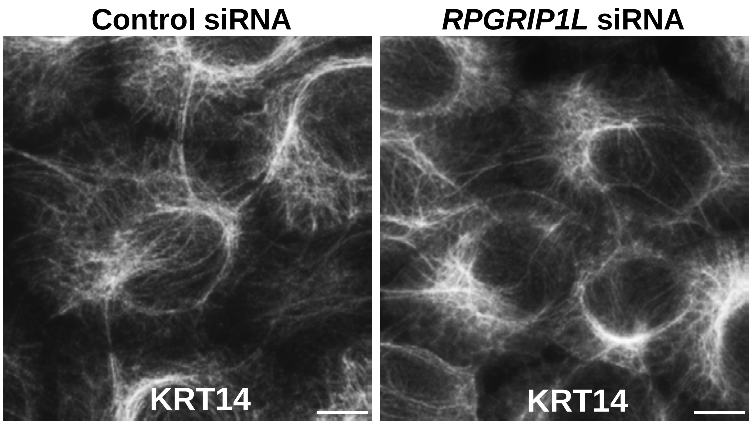

S8 Fig. Immunofluorescence labeling of keratin 14 (KRT14) in control (Control siRNA) and *RPGRIP1L*-knockdown (*RPGRIP1L* siRNA) HaCaT cells. Scale bar, 10 µm.
